# Divergent structural trajectories for pallial regionalization within reptiles reveal conserved evolutionary blueprints

**DOI:** 10.1093/cercor/bhag131

**Published:** 2026-09-16

**Authors:** Sara Jiménez, Ana Moreno-Cerdá, Juan-Antonio Moreno-Bravo, Nerea Moreno, Fernando García-Moreno

**Affiliations:** Achucarro Basque Center for Neuroscience, Scientific Park of the University of the Basque Country (UPV/EHU), Santsoena 7, Leioa 48940, Spain; Instituto de Neurociencias, Consejo Superior de Investigaciones Científicas & Universidad Miguel Hernández, Av. Don Santiago Ramón y Cajal, s/n, Sant Joan d’Alacant 03550, Spain; Instituto de Neurociencias, Consejo Superior de Investigaciones Científicas & Universidad Miguel Hernández, Av. Don Santiago Ramón y Cajal, s/n, Sant Joan d’Alacant 03550, Spain; Department of Cell Biology and Histology, Faculty of Biological Sciences, University Complutense, Calle José Antonio Novais, 12, Madrid 28040, Spain; Achucarro Basque Center for Neuroscience, Scientific Park of the University of the Basque Country (UPV/EHU), Santsoena 7, Leioa 48940, Spain; Department of Neuroscience, Faculty of Medicine and Odontology, UPV/EHU, Barrio Sarriena s/n, 48940 Leioa, Bizkaia, Spain; IKERBASQUE Foundation, María Díaz de Haro 3, 6th Floor, Bilbao 48013, Spain

**Keywords:** cortex, light sheet microscopy, neurogenesis, non-avian reptiles

## Abstract

The pallium is the evolutionary precursor of the neocortex and a central brain region underlying learning or social behavior, yet its histogenetic organization and cellular composition remain debated. Reptiles provide a key comparative framework to investigate pallial diversification and ancestral amniote traits. Here we analyzed the pallium of the turtle *Trachemys scripta elegans* and the gecko *Paroedura picta* using molecular markers, hybridization chain reaction, and 3D light-sheet fluorescence microscopy. We identify two subdivisions of the dorsal cortex defined by Satb1/Satb2 expression, consistent with patterns reported in other sauropsids. Turtles display a pronounced rostrocaudal pallial thickening, whereas in geckos this expansion is restricted rostrally, suggesting a more limited dorsal pallial enlargement in lepidosaurs. Conversely, geckos possess a nucleus sphericus absent in turtles that may correspond to the pallial amygdala described in chelonians. Other pallial territories, including medial and lateral cortical regions and the dorsal ventricular ridge, show conserved organizational features consistent with their presence in the last common ancestor of amniotes. Developmental analyses in turtles revealed apical and basal progenitors but no detectable *eomes*^+^ intermediate progenitors. These findings indicate that reptilian pallial diversification primarily reflects lineage-specific modulation of conserved neuronal transcriptional programs, providing insight into the evolutionary origins of the neocortex.

## Introduction

The most dorsal region of the vertebrate telencephalon, the pallium, is fundamental for understanding the evolution of complex cognitive functions. Across the diverse evolutionary lineages of amniotes (mammals, birds, and reptiles) this region has assumed a central role in sophisticated cognitive processes essential for survival, including learning, spatial memory, decision-making, problem-solving, emotional and social responses, as well as adaptation to novel situations (Rodríguez et al. 2002; Northcutt 2013; Medina et al. 2019; Güntürkün et al. 2021). Beyond its functional relevance, the pallium is notable for its remarkable anatomical diversity: it is organized in layers in mammals and reptiles (Nieuwenhuys et al. 1998; Nieuwenhuys et al. 2014), forms nuclear complexes in birds (Puelles 2001; Reiner et al. 2004) and certain areas of the mammalian and reptilian pallium, and exhibits simpler arrangements in amphibians and fish (Neary 1990; Mueller et al. 2011). This heterogeneity makes the pallium one of the most complex regions of the vertebrate brain, characterized by a wide variety of neuronal types, rich dynamics of cell migration, and an extensive network of connections.

A long-standing debate remains regarding the number of histogenetic domains that constitute the pallium and their homologies across vertebrate lineages (Striedter and Northcutt 2022). The tetrapartite model proposes four conserved pallial divisions in all adult vertebrates (mammals, birds and reptiles in the following examples): the medial pallium (giving rise to the hippocampus, hippocampal areas, or medial cortex), the dorsal pallium (neocortex, wulst, and dorsal cortex), the lateral pallium (claustro-insular complex, mesopallium, and lateral cortex), and the ventral pallium (pallial amygdala, nidopallium/arcopallium, and dorsal ventricular ridge) (Puelles et al. 2016; Puelles 2017). In contrast, the hexapartite model adds two further territories: the dorsolateral pallium, associated with regions such as the perirhinal cortex and caudal hyperpallium (Abellán et al. 2014; Desfilis et al. 2018), and the caudoventral pallium, linked to the posterior pallial amygdala and posterior dorsal ventricular ridge [DVR; (Medina et al. 2017; Gedman et al. 2021; Medina et al. 2022)]. In addition, an alternative model suggests that the avian pallium is organized as a continuous gradient of cell populations, arranged in functional columns, redefining the homologies between birds and mammals and suggesting that similar organizational principles may apply to other vertebrates (Jarvis et al. 2013). Although all proposals remain under discussion (Yamamoto et al. 2024), they highlight the difficulty of establishing precise homologies among lineages.

In this complex anatomical and evolutionary context, reptiles provide a valuable model for studying the processes of pallial specification and arrangement, as their three-layered structure may represent the simplest organized form of this region (Nomura et al. 2013b). In particular, from an evolutionary perspective, genetic data indicate that Testudines (turtles) and Archosauria (birds and crocodiles) share a more recent common ancestor with each other than with Lepidosauria (lizards, snakes, and tuataras) (Tzika et al. 2011; Chiari et al. 2012; Hedges 2012). According to this hypothesis, the first major divergence within reptiles occurred between lepidosaurs and the lineage that would later split into turtles and archosaurs (Hedges 2012). This intermediate phylogenetic position makes turtles a particularly valuable model for investigating the evolution of the pallium and other brain structures in amniotes. Geckos, by contrast, consistently emerge in molecular analyses as the earliest divergent branch of Squamata, that is, the most basal group among modern lizards (Vidal and Hedges 2009; Zhan et al. 2024). With this study, we aim to broaden neuroanatomical comparisons between turtles and lepidosaurs, building on existing data derived primarily from representatives of Iguania or Lacertoidea (Desfilis et al. 2018; Tosches et al. 2018; Hain et al. 2022), while addressing the lack of studies involving key lineages such as Gekkota.

The main objective of our study is to characterize and compare the genoarchitectonic organization and cellular composition of the developing and mature pallium in the turtle *Trachemys scripta elegans* and the gecko *Paroedura picta*, through a comparative approach that integrates immunohistochemistry, in situ hybridization (ISH), hybridization chain reaction (HCR), whole-mount immunolabeling and 3D light-sheet fluorescence microscopy in order to identify conserved and divergent pallial territories and neuronal types across these lineages.

Here we show that turtles and geckos share a conserved pallial organization in several domains, such as the medial, dorsomedial, and lateral cortices, as well as the DVR and the medial amygdala, supporting the presence of such territories in the last common ancestor of amniotes. However, we also identify lineage-specific features: turtles exhibit a prominent rostrocaudal pallial thickening not found in geckos, while geckos possess the nucleus sphericus, likely involved in vomeronasal processing. Moreover, we reveal differences in the structural arrangement of the dorsal cortex and in the presence of *eomes* + intermediate progenitors, which appear to be absent in reptiles. These findings provide a comparative framework to explore the evolutionary diversification of pallial domains and may shed light on the developmental origins of the neocortex.

## Materials and methods

### Animals

All animal procedures were approved by the local ethics committee and conducted under personal and project licenses in accordance with European Union regulations (Directive 2010/63/EU) and Spanish legislation (Royal Decrees 1201/2005 and 53/2013, Law 32/107).

Embryos of the red-eared slider turtle (*T. scripta elegans*) at stages 14 to 25 were obtained in collaboration with the *Centro de Conservación de Especies Dulceacuícolas de la Comunidad Valenciana* (CCEDCV) during the nesting season (May–September). Juvenile adults were obtained by incubating eggs at 28 to 29 °C and rearing hatchlings in the laboratory until the desired developmental stages were reached. Fertilized eggs of the Madagascar ground gecko (*P. picta*) were collected from a breeding colony at the Achucarro Basque Center for Neuroscience (Spain). Animals were maintained under a 12:12 h light/dark cycle (lights on at 08:00, 27 °C; lights off at 20:00, 22 °C) with ad libitum access to live crickets and water. Gecko eggs were incubated at 28 °C under low-humidity conditions until the required developmental stages were obtained.

These two non-avian reptile species were selected due to their well-characterized developmental patterns, which facilitate accurate staging during embryogenesis (Greenbaum 2002; Noro et al. 2009). In addition, the availability of a sequenced genome in *T. scripta elegans* (Schoch et al. 2020) provides an advantageous resource for gene expression studies.

### Tissue preparation for histology in sections

Embryos were anesthetized by placing the egg on ice for several minutes, whereas juvenile/adult individuals were anesthetized by intraperitoneal injection of sodium pentobarbital (50 to 100 mg/kg; Normon Labs, Madrid, Spain).

Developing stages were immersion-fixed, and juvenile adults were perfused transcardially with 4% paraformaldehyde in phosphate-buffered saline (PBS; 0.1 M, pH 7.4). In both cases, brains were post-fixed for up to 2 h and subsequently cryoprotected in 30% sucrose in PBS for 4 to 6 h at 4 °C until sinking. For immunohistochemistry, brains were embedded in 20% gelatin (OXOID, LP0021) with 30% sucrose in PBS and stored for 6 h in 4% formaldehyde at 4°C. Blocks were sectioned at 35 μm on a freezing microtome in transverse or horizontal planes. Free-floating sections were collected and rinsed in cold PBS. For classical ISH or HCR, brains were embedded in 7.5% gelatin with 30% sucrose in PBS and immediately frozen in isopentane at −80 °C. Blocks were sectioned at 35 μm on a cryostat Leica CM1950 in transverse or sagittal planes, and sections were collected on SuperFrost slides.

### Immunohistochemistry on sections

Single and combined immunohistochemical reactions were performed on free-floating sections using the primary antibodies listed in Table 1 (commercial source, immunogen information, and dilution). Sections were incubated with primary antibodies for 48 h at 4 °C, followed by incubation with the appropriate fluorophore-conjugated secondary antibodies (1:500, 90 min, room temperature): Alexa 568 goat anti-rabbit IgG (Molecular Probes, A11011), Alexa 488 goat anti-rabbit IgG (Molecular Probes, A11034), Alexa 488 goat anti-mouse IgG (Molecular Probes, A11001), and Alexa 568 goat anti-mouse IgG (Molecular Probes, A11004). All antibodies were diluted in PBS containing 0.5% Triton X-100. Sections were then rinsed in PBS supplemented with 1.5 μg/ml 4′,6-diamidino-2-phenylindole (DAPI) and coverslipped with Fluoromount aqueous mounting medium (Sigma-Aldrich; F4680).

**Table 1:** Antibodies used for immunohistochemistry. Details of primary antibodies, including immunogen, commercial supplier or source, catalog reference, and working dilution.

Name	Immunogen	Commercial supplier	Dilution
**Ctip2**	150 amino acids located at the extreme amino terminus of the protein, CTIP2 (1 to 150)	Monoclonal rabbit anti-Ctip2. Absolute antibody. Catalog reference: Ab00616–23.0	1:500
**GFAP**	GFAP isolated from cow spinal cord	Polyclonal rabbit anti-GFAP. Invitrogen. Catalog reference: PA5–16291	1:100
**Lhx2**	Amino acids: 231 to 406 of human LIM/homeobox protein Lhx2	Monoclonal mouse anti-Lhx2. Developmental Studies Hybridoma Bank. Catalog reference: 2C10	1:100
**Meis2**	Amino acids: 233 to 365 of human Meis homeobox 2	Monoclonal mouse anti-Meis2. Developmental Studies Hybridoma Bank. Catalog reference: PCRP-MEIS2-1A11	1:100
**Otp**	Amino acid sequence: RKALEHTVSMSFT of the C-terminus of OTP protein	Pickcell Laboratories, Kruislaan, Amsterdam, The Netherlands, rabbit polyclonal. RRID: AB_2315023	1:500
**PH3**	Amino acid sequence containing phosphorylated Ser 10 of Histone H3	Polyclonal rabbit anti- p-Histone H3 (Ser 10)-R. Santa Cruz. Catalog reference: sc-8656-R	1:500
**Prox1**	Synthetic peptide from the C-terminus of mouse Prox1	Polyclonal rabbit anti-Prox1. Millipore. Catalog reference: AB5475.	1:1,000
**Satb1**	Amino acids: 241 to 310 of SATB1 of human origin	Mouse monoclonal anti-Satb1. Santa Cruz. Catalog reference: sc-376096	1:100
**Satb2**	DNA-binding protein SATB2 of human origin	Rabbit monoclonal anti-SATB2. Abcam. Catalog reference: ab92446	1:1,000
**Tbr1**	Amino acids: 1 to 200 at the N-terminus of mouse TBR-1	Polyclonal rabbit anti-Tbr-1. Santa Cruz Biotechnology. Catalog reference: sc-48,816	1:500

### In situ hybridization on sections

Plasmids for ISH were linearized using the appropriate restriction enzymes (Promega, Madison, WI, United States) and served as templates for in vitro transcription with the corresponding RNA polymerases (Promega) (see Table 2 for details). Riboprobes were synthesized in the presence of digoxigenin-11-UTP (Roche Diagnostics, Mannheim, Germany).

**Table 2:** Plasmid constructs used in this study. List of genes cloned into expression plasmids, including GenBank accession numbers, sequence origin, plasmid, linearization enzymes, and polymerases used for in vitro transcription.

Gene	GenBank ref. sequence	Origin	Plasmid	Linearization enzyme and polymerase
***etv1***	XM_024107160.1	Dr. Guilles. Max-Planck-Institute for Brain Research. Frankfurt, Germany.	PCR®II	*EcoRV*/SP6
***reelin***	XM_034766147.1	Dr. Guilles. Max-Planck-Institute for Brain Research. Frankfurt, Germany.	PCR®II	*EcoRV*/SP6
***zic2***	XM_034758212.1	Dr. Guilles. Max-Planck-Institute for Brain Research. Frankfurt, Germany.	PCR®II	*SpeI*/T7

The chromogenic ISH protocol was adapted from (Ferran et al. 2015), with minor modifications for cryostat-prepared sections. Briefly, 30 to 40 μm free-floating sections were collected from fresh-frozen tissue cut on a cryostat, post-fixed in 4% paraformaldehyde, and treated with proteinase K to enhance probe penetration. Hybridization was performed overnight at 65 °C in hybridization buffer (50% formamide, 5× SSC, 50 μg/mL heparin, 250 μg/mL yeast tRNA, 5× Denhardt’s solution, 0.2% Tween-20, and 10% dextran sulfate in DEPC-treated water) containing 1 to 3 ng/μL of the digoxigenin-labeled riboprobe.

Following hybridization, sections underwent a series of stringent washes to remove unbound probe. Detection was carried out using an alkaline phosphatase-conjugated anti-digoxigenin antibody (Roche Diagnostics), and the signal was visualized with the chromogenic substrates nitro-blue tetrazolium chloride (NBT) and 5-bromo-4-chloro-3′-indolyl phosphate p-toluidine salt (BCIP). Finally, sections were mounted, dehydrated, and coverslipped for microscopic analysis.

### Hybridization chain reaction on sections

Transcript detection was performed using hybridization chain reaction (HCR v3.0, Molecular Instruments), following the manufacturer’s protocol [for further details, see (Jiménez et al. 2025)]. Tissue sections were thawed at room temperature, washed in PBS, and permeabilized with PBST (1× PBS + 0.5% Triton X-100). Sections were then incubated in pre-hybridization buffer for 30 min at 37 °C in SecureSeal™ hybridization chambers (Grace Bio-Labs) to prevent evaporation and cross-contamination. Subsequently, a mixture of DNA probes specific to the target mRNAs (see Table 3), diluted in hybridization buffer, was added and incubated overnight at 37°C.

**Table 3:** *CDS of RNA sequences targeting etv1 and eomes.* pools of split-initiator DNA probes *designed against etv1 and eomes, with GenBank accession numbers and sequence identifiers*.

Gene	GenBank ref. sequence	Amplifier	Pool sequence
***etv1***	XM_060248586.1	B3	GTCCCTGCCTCTATATCTttCGTCAAAATGAGACAGAGGCACCGT CCCCCTCTTGCATGTAGACCATGCTttCCACTCAACTTTAACCCG GTCCCTGCCTCTATATCTttAGTCTTCAGCAAGGGGCGCTGGTTG CTCTTCGTTGATATGGCGCTCCATGttCCACTCAACTTTAACCCG GTCCCTGCCTCTATATCTttGGATCACAAACAAACTTGTACACGT GGAAAGGCCATGGAGAAGAGGGCTTttCCACTCAACTTTAACCCG GTCCCTGCCTCTATATCTttCCTTCTCATAGTAATAGCGAAGTGA TCTCTCCGGCGACCTTTTGCATGATttCCACTCAACTTTAACCCG GTCCCTGCCTCTATATCTttCCTGTTTTTCTGAATCCCCCAGCGA GCTAAGTTTATCATAGTTCATAGCTttCCACTCAACTTTAACCCG GTCCCTGCCTCTATATCTttAATTCCATGCCTCGGCCAGTCCAGG GCCACCTCTTCTGGTTCAATAAGTTttCCACTCAACTTTAACCCG GTCCCTGCCTCTATATCTttGAAGAGCTACCAAAAACTGCCACAG TGAAGTGAGAGTTTGATGGATCATCttCCACTCAACTTTAACCCG GTCCCTGCCTCTATATCTttACCCTCACGGAACATTCCTGGCTCC GAGGGAGCCCCGCCGCTGATATGTTttCCACTCAACTTTAACCCG GTCCCTGCCTCTATATCTttACAACACAGGTATCATCATAAAACT TTAATATCTCCATCAAACTTCTCTGttCCACTCAACTTTAACCCG GTCCCTGCCTCTATATCTttTAACTTCTGTCCTGTTTTGGTGAGC TTGGTCCCTTTTCATACATACAACAttCCACTCAACTTTAACCCG GTCCCTGCCTCTATATCTttATGGCAGCTAGGCACTTCTGAATCA GAAGCCTTCTTGCCTCATGTAAATGttCCACTCAACTTTAACCCG GTCCCTGCCTCTATATCTttTCATAGCACTGTGTTCATATACTGG GGGGGAAGTTTTGACTGGCTGAACCttCCACTCAACTTTAACCCG GTCCCTGCCTCTATATCTttTGGAAAAGGGATGTTTGGCTCAGAC ATGGTACTCCTGCTTAAACCCTTGTttCCACTCAACTTTAACCCG GTCCCTGCCTCTATATCTttCTTGGCATTGTAGGCAAAGGAGGGA TGCCGTTGGTACATTGGGCGTCCTTttCCACTCAACTTTAACCCG GTCCCTGCCTCTATATCTttGAAATCTGTGGTCCATAGGGAAGCT AATTGCAAGGTTCTGAGAGCTGACGttCCACTCAACTTTAACCCG GTCCCTGCCTCTATATCTttATGAGCAGGAAAAACCCGGTCAGGT ATCTGGCATGGGTTGGGTTGGAGGCttCCACTCAACTTTAACCCG GTCCCTGCCTCTATATCTttTGATGCAGTGGTGAAACTGGAGTGC GGATTCTGAGCCGAGTTTGGAGATGttCCACTCAACTTTAACCCG GTCCCTGCCTCTATATCTttTTCCCACTTGTGGCTTCTGATCATA ACGGAGTTGGAGGGTTGGAGGGCCTttCCACTCAACTTTAACCCG GTCCCTGCCTCTATATCTttATAACTGAACTTGAACGGCTGTTCT ACTGACATTGTACAGGCACTTTTCCttCCACTCAACTTTAACCCG GTCCCTGCCTCTATATCTttGGACTGTGGGGTTCCTTCTTGATTT CTGCAAGCAGAGCCGAGCTCAGAACttCCACTCAACTTTAACCCG
***eomes***	XM_034760826.1	B2	CCTCGTAAATCCTCATCAaaGCCCATGCCTTTTGAAGTGTCTTTG CTAAGTAGTAGTATAGAAAGCATAGaaATCATCCAGTAAACCGCC CCTCGTAAATCCTCATCAaaTTCATTGTTGGAGAATTTTCATTGC TAGTCCTCAGCATTCATGTCTTCACaaATCATCCAGTAAACCGCC CCTCGTAAATCCTCATCAaaTACAAGCACTAGTGTAGACACCAGA AAGTGCTAGGAGAGAGTCGTCTTCTaaATCATCCAGTAAACCGCC CCTCGTAAATCCTCATCAaaTGGGGTCTCTATCCAGGATGAGCCG GTTTGAATCTAGAGATTTTATGGAAaaATCATCCAGTAAACCGCC CCTCGTAAATCCTCATCAaaTGGTCTTCAGAGAAACCCGGGGGAC TCTTCTTTCACCTTCTCCTTGGGAAaaATCATCCAGTAAACCGCC CCTCGTAAATCCTCATCAaaCCATTTTTCTCTGATAAGAACCTCT TTCGGGAGGTCCAAGGTAACCCAGTaaATCATCCAGTAAACCGCC CCTCGTAAATCCTCATCAaaTGGATCAGGATAATATCCCAAAGCA TCCCCACCCTGCCATGGAAGGGAATaaATCATCCAGTAAACCGCC CCTCGTAAATCCTCATCAaaCCATAGGGGAGCAAGGTGCTAGGAG GATGTCTGCAAGGGAAGGGATTTAAaaATCATCCAGTAAACCGCC CCTCGTAAATCCTCATCAaaGTTTGTTGGTACCAGGCTGCTGCAC ATTCTGTTTCATAGGAGTTCATGTCaaATCATCCAGTAAACCGCC CCTCGTAAATCCTCATCAaaGGCCACCTCTTCATTCTGCTGAGGA AGTAACAAACCATCTCTGGGGAGGAaaATCATCCAGTAAACCGCC CCTCGTAAATCCTCATCAaaCTCTCACCATTGTAAAATCTGGCTG
			22 AGAAGGCCATTTGTCTGAGGTACAGaaATCATCCAGTAAACCGC 23 CCTCGTAAATCCTCATCAaaAGAATGGTTGTACGCTGTACCGGGC 24 GCAAGTTGTTGACAAACTGTTCCTGaaATCATCCAGTAAACCGCC 25 CCTCGTAAATCCTCATCAaaATCCGTGGGAGATGGAGTTAATCTG 26 TGGGACAATCTGGTGGGATCTAGGAaaATCATCCAGTAAACCGCC 27 CCTCGTAAATCCTCATCAaaTAGTTGTCCCTGAAGCCTTTTGCAA 28 TTTTCTGAAGCTGTGTACATGGAATaaATCATCCAGTAAACCGCC 29 CCTCGTAAATCCTCATCAaaTATCAGTGTTTTGATATGCAGTAAC 30 GATTATGGTCAATTTTAAGCTGAGTaaATCATCCAGTAAACCGCC 31 CCTCGTAAATCCTCATCAaaCGTCTGCGTCTTTGAAGAGTCATTC 32 TATGAACTGGGTTTCCGGGAAAACAaaATCATCCAGTAAACCGCC 33 CCTCGTAAATCCTCATCAaaACAATATGAAGTCGAGGTTGGTATT 34 TCTTCAACCCCATCTTCAGTCACTTaaATCATCCAGTAAACCGCC 35 CCTCGTAAATCCTCATCAaaTATTATTATTGTTAGCACCTTTGTT 36 GTAGAGACTGAAGTACTATCATCTGaaATCATCCAGTAAACCGCC 37 CCTCGTAAATCCTCATCAaaCTCCTGCCTCATCCAGTGAGCCCCA 38 GGTCAGTTTTAGCTTTCCAAAAGAGaaATCATCCAGTAAACCGCC 39 CCTCGTAAATCCTCATCAaaTTGTTGCCTTGCATGTTGTTGTCCG 40 TTCGGGGACTCAGGGTGAACATACAaaATCATCCAGTAAACCGCC 41 CCTCGTAAATCCTCATCAaaCGGCCAGCACCACCTCCACGAACAC 42 CCCCCTGGAAGCGCCAGTGGTTGGGaaATCATCCAGTAAACCGCC 43 CCTCGTAAATCCTCATCAaaGATGTTGAAGCTCAGGAAGGGAAAC 44 GTAATGCGCCGTGGGGTTGAGGCCGaaATCATCCAGTAAACCGCC 45 CCTCGTAAATCCTCATCAaaTCCGTCTGGTGCCGGTGGAACTTGA 46 CGCCTGCCCTGCTTGGTGATGATCAaaATCATCCAGTAAACCGCC 47 CCTCGTAAATCCTCATCAaaGCGCCCGGAGCCCTGGCCCCGAGCC 48 AGAGCGGGCGGTTGCAGAGGAAGACaaATCATCCAGTAAACCGCC 49 CCTCGTAAATCCTCATCAaaGGAGCCCGCCGCCGGGTAGGGGCTG 50 CACGGCTAAGGCGCCCAGCCCGCCGaaATCATCCAGTAAACCGCC 51 CCTCGTAAATCCTCATCAaaTGGTATCCGCCGCCGCCCCCGAACT 52 AGGGGCCCCGCCGCCCCTTGGCCATaaATCATCCAGTAAACCGCC 53 CCTCGTAAATCCTCATCAaaGGTAGCGCGCCCCGCCGGGCGATTG 54 CCCCCGGGGACAGCATGGAGCCGTAaaATCATCCAGTAAACCGCC 55 CCTCGTAAATCCTCATCAaaGGGGAAGAGCGAGCAGGGCGCGCCC 56 GACGGAGCCGTGCTGCGCCGCCGGGaaATCATCCAGTAAACCGCC 57 CCTCGTAAATCCTCATCAaaAGGTAGTAGCGCTCCGGGCTCAGGC 58 TCAGCCCCCTGCGGCCCGGGGGACTaaATCATCCAGTAAACCGCC 59 CCTCGTAAATCCTCATCAaaGGGGCAGCTCGGCCTCTCCGCAAGG 60 CCAGTGGGTAGCGGGCAGCGGCGGCaaATCATCCAGTAAACCGCC 61 CCTCGTAAATCCTCATCAaaCTTGGAGCCCGCGACGGGGAAGGGC 62 CGCCTTGCGGCCGTCCGGGGCGCCCaaATCATCCAGTAAACCGCC 63 CCTCGTAAATCCTCATCAaaGGCCCGGCGCCGCCGCACTTCTTCG 64 CCAGCCTCCGCCTCGCTCAGCATGCaaATCATCCAGTAAACCGCC 65 CCTCGTAAATCCTCATCAaaATCCCGAGCTGGGCTGGCTGCGGTG 66 CCTTGTCCAAGTCCAGGCGCTGCGGaaATCATCCAGTAAACCGCC

Following hybridization, sections were washed with a decreasing gradient of wash buffer and SSC, and then pre-incubated in amplification buffer. Fluorescent hairpins (h1 and h2) were snap-cooled by heating to 95°C, followed by immediate cooling on ice and equilibration to room temperature in the dark. The hairpins were then added to the sections to allow in situ signal amplification via polymerization of hybridization chains, incubated overnight at room temperature. Finally, the SecureSeal™ chambers were removed, and the sections were washed in SSCT. Samples were either processed for subsequent immunostaining or mounted using mounting medium (Sigma-Aldrich; F4680).

### Neurobiotin tracer injections

Under deep anesthesia, animals were transcardially perfused with 250 mL of ice-cold Ringer’s solution (100 mM NaCl, 400 mM NaHCO₃, 60 mM KCl, 260 mM CaCl₂, 160 mM MgCl₂, 11 mM glucose; Merck), which had been oxygenated with carbogen (95% O₂, 5% CO₂) to a pH of 7.3. Brains were then carefully dissected and transferred to fresh oxygenated Ringer’s solution at 4 °C.

The neuronal tracer Neurobiotin® (Vector Laboratories; SP1120) was injected in vitro into the ventral portion of the olfactory bulb (OB) of the isolated brain. After injection, brains were maintained at 4 °C for 24 to 48 h in continuously oxygenated Ringer’s solution to allow for tracer transport. Following incubation, brains were fixed in 4% paraformaldehyde (PFA) in 0.1 M PBS for 24 h at 4 °C. After fixation, tissue was blocked in gelatin and sectioned. Neurobiotin labeling was visualized using Alexa Fluor 594-conjugated streptavidin (1:500; Molecular Probes).

### Tissue clearing by 3Disco and visualization of wholemount samples

Tissues were processed following the iDISCO+ (Belle et al. 2014; Renier et al. 2016; Belle et al. 2017), with specific modifications to optimize whole-mount immunolabeling. Briefly, samples were dehydrated through a graded methanol/PBS series (20% to 100%) and subsequently treated with dichloromethane to clear the brain and hydrogen peroxide in methanol to reduce autofluorescence.

After rehydration, tissues were permeabilized in PTx.2 solution supplemented with DMSO and glycine and then blocked using donkey serum and DMSO. Primary antibody incubations were carried out at 37 °C for 3 to 7 days, depending on sample size, using the same antibody concentrations as for histological sections, followed by extensive washes in PTwH buffer. Secondary antibody incubation (1:500 dilution) was performed under the same conditions, with samples protected from light.

Finally, tissues underwent progressive methanol dehydration and clearing with dichloromethane and dibenzyl ether (DBE). Cleared samples were stored in DBE at room temperature in the dark until imaging. Three-dimensional imaging was performed with a light-sheet fluorescence microscope (Ultramicroscope II, LaVision BioTec, Bielefeld, Germany), using ethyl cinnamate (ECi) as the refractive index matching solution.

### Imaging and image processing

Bright-field images were acquired using an Olympus BX51 microscope equipped with a digital camera (Olympus DP72). Fluorescence immunostaining images were collected either with a Zeiss LSM 710 confocal microscope (Carl Zeiss Microimaging, Germany) or a 3DHistech Panoramic Midi II digital slide scanner (3DHistech, Hungary), using a 20× air objective. For images obtained from the same brain, acquisition parameters (laser power, gain, and detection wavelengths) were kept constant, while adjustments were made only when acquiring new slides. Large brain sections were imaged using tile-scan acquisition. Images obtained with the digital slide scanner were acquired in single-layer mode with the 20× objective, sequentially collecting the signal from individual fluorophores. All images were processed using FIJI (ImageJ), and further adjustments were made in Adobe Photoshop CS6 (Adobe Systems, San Jose, CA). Final figures were assembled in Canvas X Draw (ACD Systems, Canada).

In the case of images acquired through *light sheet* fluorescence microscopy, image processing, 3D reconstructions, and videos were generated using Imaris ×64 software (version 10.0, Bitplane). Image stacks were first converted to Imaris format (.ims) using ImarisFileConverter, and 3D reconstruction was performed with the “Volume Rendering” function. Optical sections were obtained using the “Orthoslicer” tool, while specific tissue regions were manually isolated with the “Surface” tool by applying the “Mask” option. Three-dimensional images and animations were generated using the “Snapshot” and “Animation” tools, respectively. Final video reconstruction and editing from.mp4 file series were performed using Microsoft Clipchamp (online version).

## Results

### Organization of the telencephalon in turtle and gecko during development and adulthood

**Figure 1:**
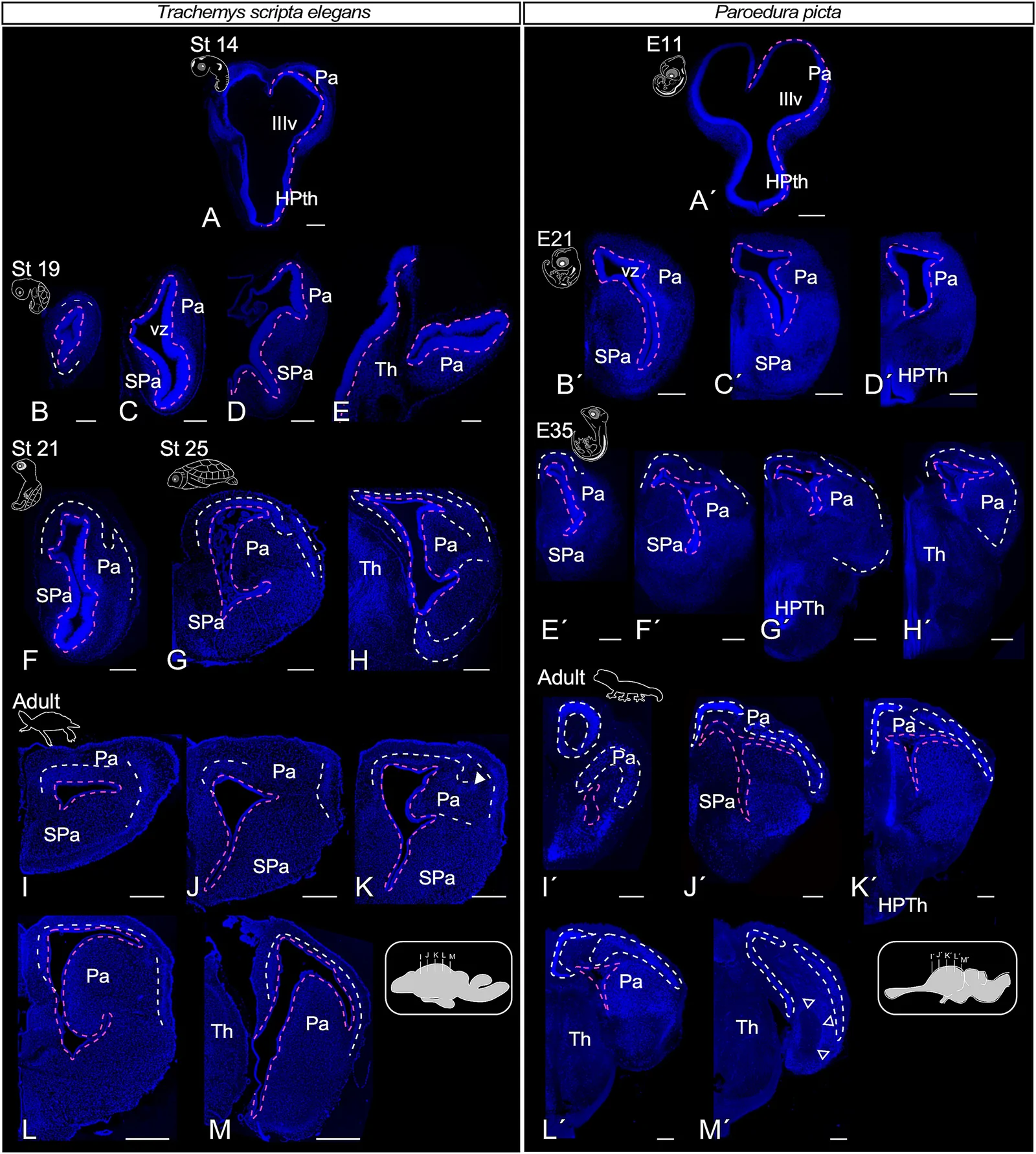
Rostrocaudal organization of the forebrain in turtle and gecko during development and adulthood. Nuclear staining with DAPI in representative coronal sections of the forebrain of *Trachemys scripta elegans* (turtle) and *Paroedura picta* (gecko) at embryonic and adult stages. Sections are arranged in rostrocaudal order to compare the general organization of the pallium, subpallium, and adjacent diencephalic regions. (A, A’) Early stages of turtle (St14) and gecko (E11), showing a broad ventricular zone and a poorly developed pallial mantle. (B–H, B’–H’) Intermediate (St19/E21) and advanced embryonic stages (St21–25/E35), showing the progressive evagination of the telencephalic vesicles, the relative reduction of the ventricular zone, and the emergence of a laminar organization in pallial regions. (I–M, I’–M’) Adult brains, showing the rostrocaudal organization of the pallium in both species. In turtle, a prominent pallial thickening is observed at anterior and intermediate levels, whereas in gecko the cortical regions display a more compact and dorsally shifted arrangement. Magenta lines delimit the ventricular zone. White lines indicate the laminar organization of pallial regions. Abbreviations: HPTh, hypothalamus; IIIv, third ventricle; Pa, pallium; SPa, subpallium; Th, thalamus; VZ, ventricular zone. Scale bars: A, A’ = 500 μm; B–M, B’–M’ = 200 μm.

To establish an anatomical reference framework, we analyzed the organization of the telencephalon at different embryonic stages and in adulthood in *T. scripta elegans* and *P. picta* using DAPI staining (Fig. 1). Nuclear labeling allowed the delineation of the main telencephalic subdivisions, including the pallium (Pa) and subpallium (SPa), as well as adjacent regions such as the thalamus (Th) and hypothalamus (HPTh) in both species.

At the earliest stages tested (turtle St14/gecko E11; Fig. 1A–A’), the pallium formed part of a continuous neurogenic epithelium within the telencephalon. It was mainly composed of a thick ventricular zone (VZ), probably highly proliferative, and a thin mantle. At intermediate stages (St19/E21; Fig. 1B–D’), the evagination of the telencephalic vesicles became evident, accompanied by a progressive thickening of the pallial mantle, while the ventricular zone remained relatively wide. At this stage, the pallium still lacked defined laminar or nuclear arrangements. The laminar organization in medial, dorsomedial, dorsal and lateral cortex (highlighted by white outlines) became apparent at later stages (St21–25/E35; Fig. 1F–H’), coinciding with the progressive narrowing of the ventricular zone (highlighted in magenta dashed lines). Based on these morphological similarities, we considered the developmental stages compared across species to be equivalent in broad pallial anatomical terms.

In juveniles/adults (Fig. 1I–M’), marked differences emerge between turtle and gecko. In turtles, the pallium exhibits a prominent thickening that extends dorsolaterally to reach the surface of the lateral cortex (Fig. 1K, white arrowhead), forming a large nuclear structure characteristic of chelonians. In geckos, this structure is less pronounced; instead, caudally it was observed a nucleus with a characteristic laminar arrangement (Fig. 1M’, empty arrowheads), absent in chelonians. Moreover, geckos displayed smaller ventricles, greater overlap between cortical layers, and a dorsal shift of the layers in the medial pallium compared to turtles, in addition to a higher degree of cellular compactness.

These observations revealed the conservation of a general pattern of pallial regionalization during development in both species, while also highlighting significant differences in the nuclear and laminar organization of the adult pallium.

### Conserved molecular and cellular features of the medial pallium in turtle and gecko

**Figure 2:**
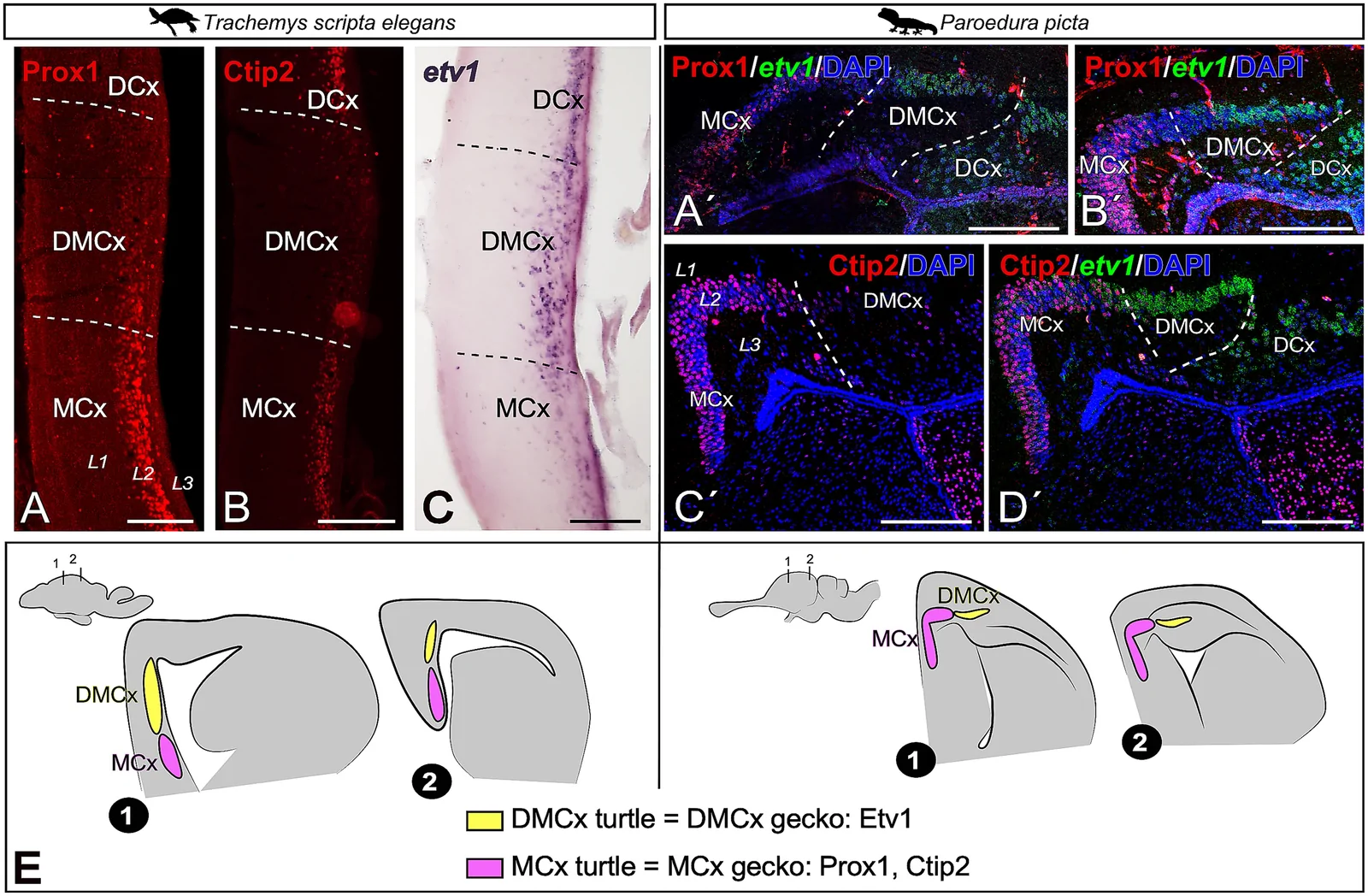
Molecular characterization of the medial pallium in turtle and gecko. Expression of Prox1, Ctip2, and *etv1* in the medial pallium of *Trachemys scripta elegans* and *Paroedura picta*, analyzed by immunohistochemistry, in situ hybridization, and HCR. These markers delineate the medial cortex (MCx) and the dorsomedial cortex (DMCx) in both species. (A–C) In turtle, Prox1 and Ctip2 identify the MCx, whereas *etv1*, together with low or absent Ctip2 levels, delineates the DMCx. (A’–D’) In gecko, the combination of Prox1, Ctip2, and *etv1* defines territories equivalent to the MCx and DMCx, with a more compact and dorsally shifted arrangement. (E) Schematic summary of the correspondence between the medial domains identified in turtle and gecko. White dashed lines delimit the medial pallial territories defined by the combination of markers. Abbreviations: DCx, dorsal cortex; DMCx, dorsomedial cortex; MCx, medial cortex. Scale bar: 200 μm.

To investigate the regional identity of the medial pallium (MP), we analyzed the expression of molecular markers by immunohistochemistry, ISH, and HCR (Fig. 2). The analyzed markers (Prox1, Ctip2 and *etv1*) were selected based on their previously documented expression in the hippocampal formation of birds, the hippocampal regions of mammals, and the medial cortices of other lepidosaur and crocodilian species (Lavado et al. 2010; Gupta et al. 2012; Briscoe and Ragsdale 2018b; Tosches et al. 2018). In both species, Prox1 and Ctip2 consistently identified the medial cortex (MCx; Fig. 2A, B, A’–D’), whereas the combined expression of *etv1* and Ctip2 helped delineate the dorsomedial cortex (DMCx; Fig. 2C, A’, B’, D’). These complementary patterns clearly defined the boundaries between the MCx, the DMCx, and the adjacent dorsal pallial regions.

In turtle, the combined expression of Prox1, Ctip2, Satb1, and *etv1* allowed us to identify the medial and dorsomedial cortical territories, as well as to distinguish them from the dorsal cortex (Fig. 2A–C). The MCx was defined by prominent Prox1 and Ctip2 expression (Fig. 2A, B). By contrast, the DMCx was identified as a territory with low or absent Ctip2 expression, interposed between the medial and dorsal cortical domains, both of which are Ctip2^+^. In addition, the DMCx showed *etv1* expression, which was absent from the MCx but also detectable in the aDCx (Fig. 2C). In both MCx and DMCx, three layers were distinguished: layer 3 (L3), with lower cell density and more dispersed cells; layer 2 (L2), showing the highest degree of cellular compactness (Fig. 2A, C’); and layer 1 (L1). In geckos, L1 was markedly wider than in turtles, which could be explained by an extended radial migration of neurons away from the ventricle, in contrast to turtles, on which they appear to remain adjacent to it after neurogenesis (compare Fig. 2B and C). In addition, cells in L2 were more compact in geckos than in turtles (compare Fig. 2C and C’). Furthermore, the DMCx exhibited a dorsal shift in geckos in contrast to turtles, where this structure remained localized along the medial axis (Fig. 2C, D’). In both species, Prox1 and Ctip2 were primarily expressed in layer L2 of the MCx (Fig. 2A, A’–D’), whereas *etv1* showed strong labeling in layer L2 of the DMCx (Fig. 2C, A’–D’). Notably, no rostrocaudal variations were observed in the expression patterns of these markers in either species.

The topological similarities of these expression profiles across the MCx and DMCx domains in both species provide strong evidence for a conserved molecular architecture of the MP in non-avian reptiles (Fig. 2E).

### Regional organization of the dorsal cortex and pallial thickening in turtle and gecko

**Figure 3:**
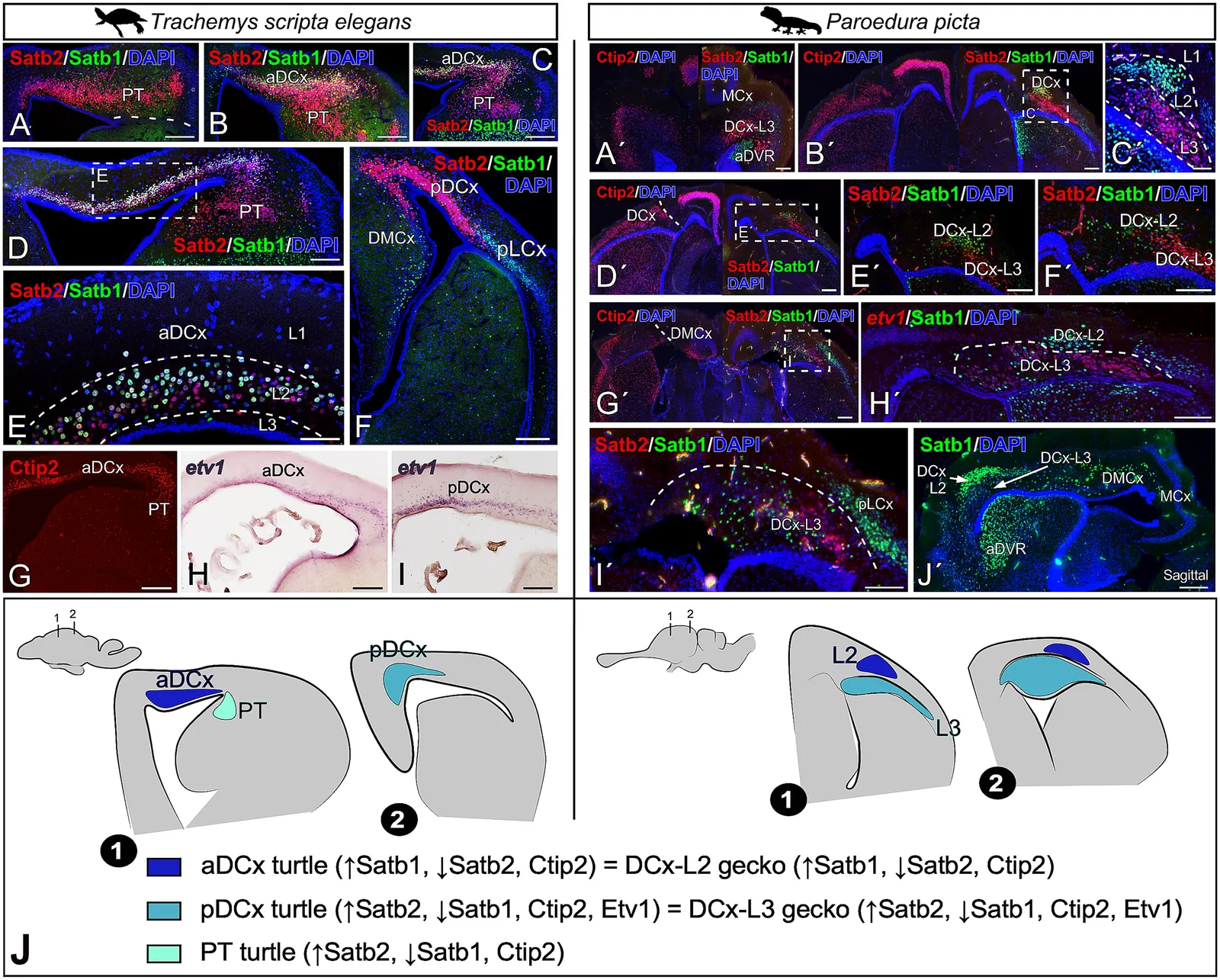
Molecular organization of the dorsal pallium in turtle and gecko. Expression of Satb1, Satb2, Ctip2, and *etv1* in the dorsal pallium of *Trachemys scripta elegans* and *Paroedura picta*. The panels compare the rostrocaudal organization of the dorsal cortex in turtle with the laminar organization of the dorsal cortex in gecko. (A–I) In turtle, the rostrocaudal series shows the pallial thickening (PT) at anterior and intermediate levels, together with the anterior dorsal cortex (aDCx) and the posterior dorsal cortex (pDCx). The PT contains a prominent Satb2^+^ population, the aDCx is characterized by a Satb1^+^/Ctip2^+^ band, and the pDCx shows higher expression of Satb2 and *etv1* together with lower expression of Satb1. (A’–I’) In gecko, comparable molecular populations are arranged as layers within the dorsal cortex. DCx-L2 shows high expression of Satb1 and Ctip2, whereas DCx-L3 shows higher expression of Satb2 and *etv1*. (J’) Sagittal section used to evaluate the anteroposterior continuity of dorsal domains. In gecko, the region topologically comparable to the PT described in other reptiles corresponds, with the markers analyzed here, to a subdivision of the dorsal cortex rather than to an independent nuclear domain. (J) Schematic summary of the proposed correspondence between aDCx/pDCx in turtle and DCx-L2/DCx-L3 in gecko. White dashed lines indicate the boundaries between dorsal domains. Abbreviations: aDCx, anterior dorsal cortex; DCx, dorsal cortex; DCx-L2/3, layers 2 and 3 of the dorsal cortex; pDCx, posterior dorsal cortex; PT, pallial thickening. Scale bars: A–D, G–I, A’, B’, D’–J’ = 100 μm; E, C’ = 50 μm; F = 200 μm.

To characterize the dorsal-most region of the pallium, we analyzed the expression of the cortical markers Satb1, Satb2, Ctip2, and *etv1* (Fig. 3). These markers, and their specific combinatorial expression patterns, have been previously identified in the dorsal pallium (DP) of birds, mammals, crocodilians, and the lizard *Pogona* (Suzuki Ikuo et al. 2012; Briscoe and Ragsdale 2018b; Nomura et al. 2018; Tosches et al. 2018), supporting their value as conserved indicators of dorsal pallial organization across amniotes. In turtles, the dorsal cortex (DCx) was not clearly identified at the most anterior levels of the telencephalon. At these levels, the pallial region was mainly occupied by the pallial thickening (PT), which extended across the dorsal portion of the pallium and into its lateral aspect (Fig. 3A). At slightly more caudal levels, the anterior dorsal cortex (aDCx) became recognizable by the emergence of a Satb1^+^ band (Fig. 3B). However, this Satb1^+^ domain was not completely segregated from Satb2^+^ cells, since Satb2^+^ cells were also present within or intermingled with the Satb1^+^ aDCx population (Fig. 3E). In addition, a prominent Satb2^+^ population was located beneath the Satb1^+^ band, which we interpret as corresponding mainly to the PT. This PT progressively shifted toward a deeper position at slightly more caudal levels, forming a non-laminar domain adjacent to the ventricle and dorsal to the DVR (Fig. 3C, D). This interpretation is consistent with previous transcriptomic and anatomical studies showing that the turtle PT is a distinct pallial domain enriched in markers such as Crhbp, Lgals1, Adarb2, and Pou3f4, whereas Satb1 is associated with the aDCx rather than the PT (Tosches et al. 2018; Norimoto et al. 2020).

The aDCx extended into anteromedial regions and was characterized by strong Satb1 and Ctip2 expression and a few scattered *etv1*+ cells. Satb2 expression in this territory was comparatively lower than in the PT and pDCx, although Satb2^+^ cells were still present within the aDCx, including cells intermingled with the Satb1^+^ population (Fig. 3D, E, G, H and Video S1). At more caudal levels, the characteristic nuclear morphology of the PT was no longer evident, and the posterior dorsal cortex (pDCx) became the predominant dorsal pallial domain. This subdivision displayed a molecular profile distinct from that of the aDCx, with prominent Satb2 and *etv1* expression, together with reduced Satb1 levels (Fig. 3F, I and Video S1). Thus, the turtle DP does not show a simple laminar Satb1/Satb2 arrangement; rather, Satb1^+^ and Satb2^+^ populations are redistributed along the rostrocaudal axis, with Satb2^+^ cells mainly associated with the PT rostrally and with the pDCx caudally.

In geckos, an equivalent molecular organization was observed, although it was structured as laminar differences within the DCx. The most notable feature was the absence of a territory comparable to the turtle PT. At anterior levels, layer 3 of the DCx (DCx-L3) was already present and extended toward more posterior regions. This layer showed higher Satb2 and *etv1* expression, together with reduced Satb1, paralleling the molecular profile of the turtle pDCx (Fig. 3A’–I’ and Video S2). By contrast, layer 2 of the DCx (DCx-L2) was characterized by high Satb1 and Ctip2 expression, together with low Satb2 and *etv1* expression, making it molecularly comparable to the turtle aDCx (Fig. 3A’–I’ and Video S2). These molecular correspondences suggest that the anteroposterior domains identified in turtle (aDCx/pDCx) are molecularly equivalent to the laminar subdivisions observed in gecko (DCx-L2/DCx-L3). By contrast, we found no evidence of a PT-equivalent territory in gecko with the markers analyzed. This conclusion is supported by both coronal and sagittal material. In coronal sections, direct comparison between turtle and gecko revealed a prominent PT in turtle but no comparable nuclear domain in gecko (Fig. S1). In sagittal sections, the region occupying a position comparable to the PT described in other gecko species (Puelles et al. 2017) corresponds, in our material, to DCx-L2, characterized by Satb1 expression, rather than to an independent PT-like domain (Fig. 3J’). Thus, the territory interpreted as PT may reflect, at least in part, a cytoarchitectonically distinct portion of the dorsal cortex, rather than a separate molecularly defined PT-equivalent domain.

### Molecular delineation of the lateral cortex in turtle and gecko

**Figure 4:**
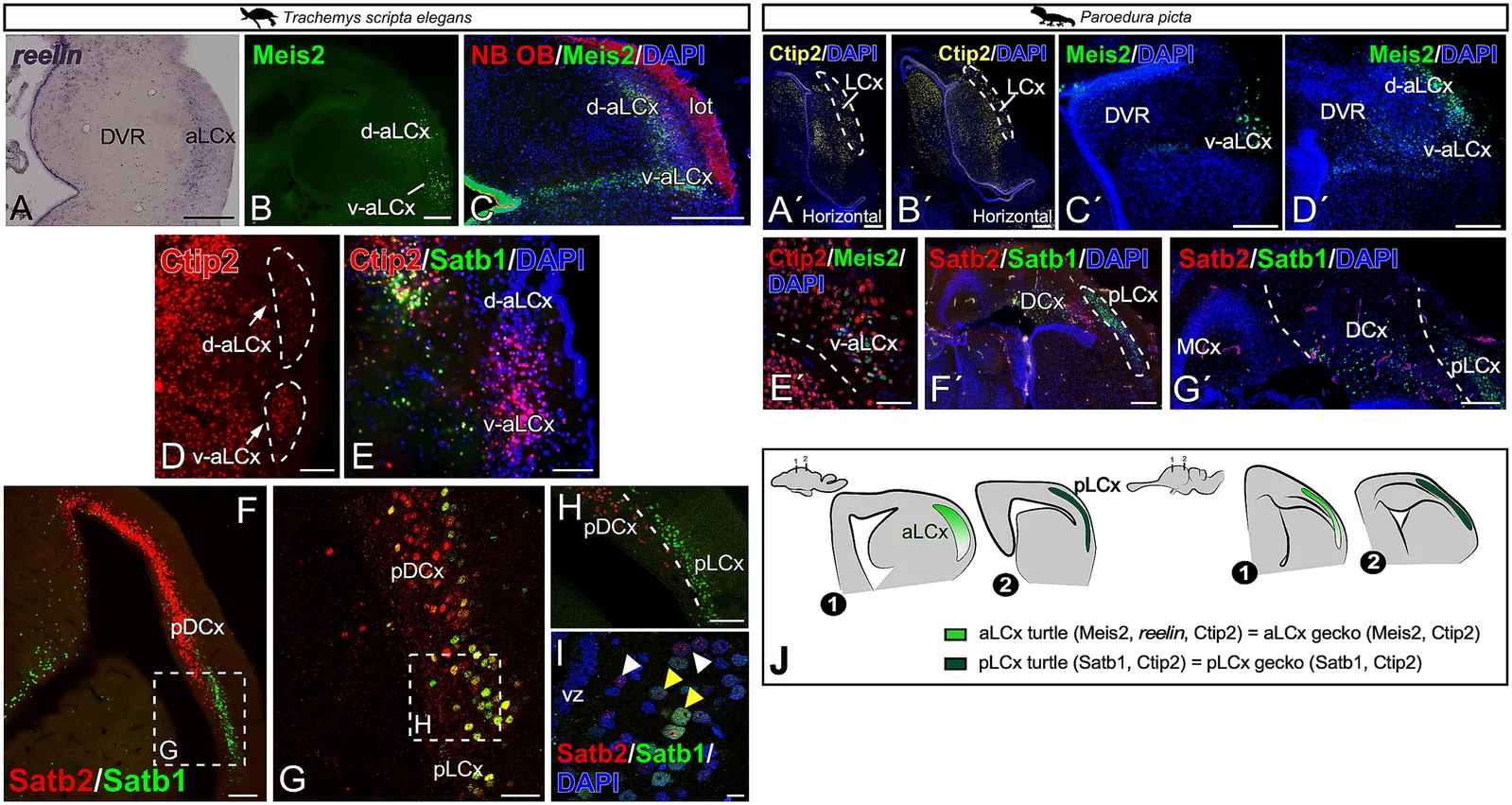
Molecular mapping of the lateral pallium in turtle and gecko. Expression of Meis2, *reelin*, Ctip2, Satb1, and Satb2 in the lateral pallium of *Trachemys scripta elegans* and *Paroedura picta*. The panels show the organization of the anterior lateral cortex (aLCx) and posterior lateral cortex (pLCx) in both species. (A–E) In turtle, the aLCx is identified by the expression of Meis2, *reelin*, and Ctip2. This domain is subdivided into a dorsal portion (d-aLCx) and a ventral portion (v-aLCx). (C) Anterograde tracing with Neurobiotin from the olfactory bulb, showing projections to the lateral pallium. (D–I) In turtle, caudal levels show the pLCx, characterized by Satb1^+^ cells and by its topological relationship with the pDCx. (A’–G’) In gecko, domains comparable to the aLCx and pLCx are identified. The aLCx expresses Meis2 and Ctip2, whereas the pLCx contains Satb1^+^ cells adjacent to the dorsal cortex. (J) Schematic summary of the organization of the lateral pallium in turtle and gecko. White dashed lines delimit the lateral territories. Abbreviations: aLCx, anterior lateral cortex; d-aLCx, dorsal portion of the aLCx; DVR, dorsal ventricular ridge; LCx, lateral cortex; NB, Neurobiotin; OB, olfactory bulb; pLCx, posterior lateral cortex; v-aLCx, ventral portion of the aLCx; VZ, ventricular zone. Scale bars: A’, B’ = 500 μm; A–C = 200 μm; D–H, C’, D’, F’, G’ = 100 μm; I, E’ = 50 μm.

To better understand the molecular identity and potential functional roles of the lateral pallium (LP), we selected a set of markers previously associated with both olfactory processing and pallial regionalization. In mammals, *reelin* and *Meis2* are prominently expressed in olfactory-related structures, including the piriform and entorhinal cortices (Stranahan et al. 2011; Agoston et al. 2014). Moreover, *reelin* expression has been documented in both the OB and the lateral cortex (LCx) of reptiles (Goffinet et al. 1999; Tosches et al. 2018). In parallel, Satb1, Satb2, and Ctip2 have been implicated in laminar organization and the definition of pallial domains across multiple vertebrate lineages, including reptiles and birds (Briscoe et al. 2018; Briscoe and Ragsdale 2018b). Given the proposed homology between the LCx and the olfactory pallium of mammals, these markers provide a robust framework for analyzing the molecular architecture and evolutionary conservation of this region. Based on these considerations, we examined the organization of the LCx by analyzing the expression of *reelin*, Meis2, Satb1, Satb2, and Ctip2 (Fig. 4).

In turtles, the LCx exhibited a clear subdivision into anterior and posterior domains. The anterior lateral cortex (aLCx) was defined by the expression of *reelin*, Meis2, and Ctip2 (Fig. 4A–E; Video S1, S3). In this domain, two territories were identified: a dorsal portion (d-aLCx) and a ventral portion (v-aLCx). The presence of Meis2+ and Ctip2+ cells was observed in both territories; however, the v-aLCx exhibited a higher concentration of cells expressing these markers (Fig. 4B–E). Despite these differences in cell density, projections from the OB reached both portions of the aLCx, as demonstrated by anterograde neurobiotin tracer analysis (Fig. 4C), thereby supporting its involvement in olfactory processing. In contrast, the posterior lateral cortex (pLCx) was characterized by an enrichment of Satb1+ cells, absent in the aLCx, and a reduction in Meis2 expression. At most caudal levels, a portion of the pLCx overlapped with the pDCx, lying dorsal to it (Fig. 4D–F). Moreover, some cells within the pLCx showed Satb1/Satb2 co-expression, in contrast to the pDCx cells, which expressed only Satb2 (Fig. 4G–I).

A comparable pattern was identified in geckos. The aLCx was likewise defined by Meis2 and Ctip2 expression and was further subdivided into d-aLCx and v-aLCx domains, similarly to those described in turtles (Fig. 4A’–E’; Video S2, S4). The pLCx also displayed a molecular profile similar to that observed in turtles, characterized by an enrichment of Satb1^+^ cells adjacent to the DCx (Fig. 4F’, G’; see also Fig. 3H’ and J’) thereby confirming the similarity of marker expression in these territories.

These findings highlighted a conserved organization of the lateral pallial region in these reptilian species, structured into anterior (d-aLCx and v-aLCx) and posterior (pLCx) domains, with comparable molecular profiles in turtle and gecko (Fig. 4J).

### Molecular characterization of the ventral pallial domain

**Figure 5:**
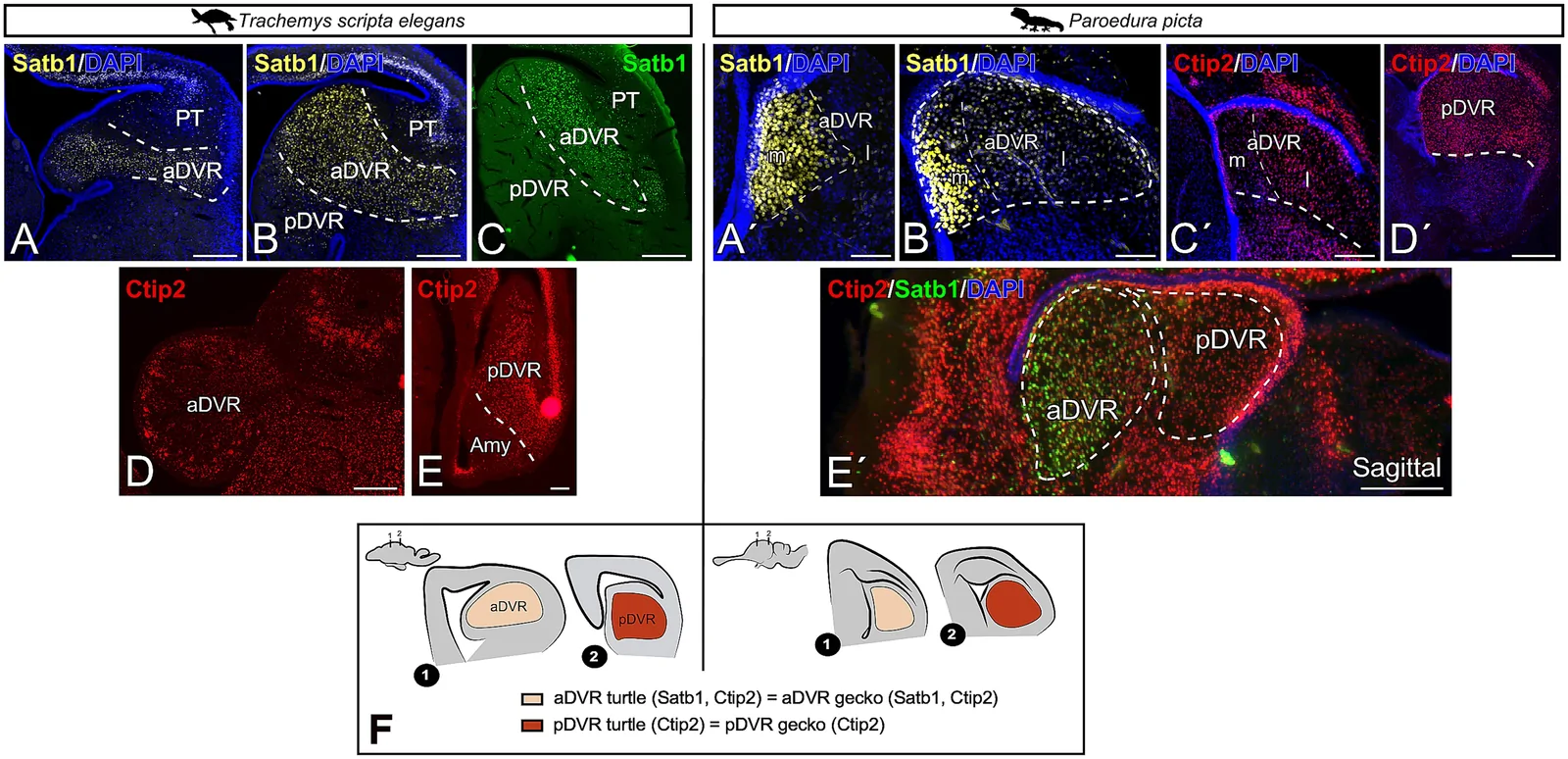
Molecular delineation of the ventral pallium in turtle and gecko. Expression of Satb1 and Ctip2 in the ventral pallium of *Trachemys scripta elegans* and *Paroedura picta*. The combination of these markers delineates the dorsal ventricular ridge (DVR) and allows its anterior and posterior subdivisions to be distinguished. (A–E) In turtle, the DVR appears as a well-delimited nuclear domain. The anterior DVR (aDVR) expresses Satb1 and Ctip2 and is located ventral to the PT and adjacent to the lateral cortex. At more caudal levels, the posterior DVR (pDVR) mainly expresses Ctip2 and occupies a larger extent of the ventral pallium. (A’–E’) In gecko, the DVR occupies a comparable topological position. The aDVR shows co-expression of Satb1 and Ctip2, with Satb1^+^ cells preferentially concentrated in periventricular positions, whereas the pDVR mainly expresses Ctip2. (F) Schematic summary of DVR organization in both species. White dashed lines delimit the ventral pallium and its subdivisions. Abbreviations: aDVR, anterior portion of the DVR; DVR, dorsal ventricular ridge; LCx, lateral cortex; pDVR, posterior portion of the DVR; PT, pallial thickening; VP, ventral pallium. Scale bar: 200 μm.

The ventral pallium (VP) is one of the most anatomically diverse regions of the vertebrate telencephalon. In reptiles, this domain gives rise to the DVR, a large nuclear structure proposed to be homologous to components of the mammalian amygdaloid complex (Medina et al. 2017; Puelles 2017; Desfilis et al. 2018). Unlike other pallial subdivisions, which typically exhibit a layered organization, the DVR is characterized by its non-laminar nuclear architecture. To delineate this region, we focused on the expression of Satb1 and Ctip2 (Fig. 5), two transcription factors previously associated with ventral pallial territories and the DVR in reptiles and birds (Briscoe and Ragsdale 2018a; Tosches et al. 2018; Rueda-Alaña et al. 2025).

In *T. scripta elegans*, the DVR was defined as a well-delimited domain. The anterior portion (aDVR) was characterized by Satb1 and Ctip2 expression and exhibited clear boundaries with the PT and LCx (Fig. 5A–D; Video S1). At those anterior levels, the aDVR was comparatively smaller, due to the prominence of the PT, which occupies most of the dorsoventral extent. However, along the caudal axis, the DVR progressively expanded radially from the ventricular zone to reach the surface, coinciding with the regression of the PT (compare Fig. 5A and B). A second DVR subdivision, the posterior DVR (pDVR), was defined by the exclusive expression Ctip2. It initially appeared ventral to the aDVR (Fig. 5B, C) and, at more caudal levels, occupied nearly the entire VP, except for a small domain corresponding to the amygdala (Amy) (Fig. 5E; Video S3).

Similarly, in *P. picta* the DVR displayed a comparable topological position and molecular profile. The aDVR exhibited co-expression of Satb1 and Ctip2 (Fig. 5A’, C’; Video S4), although Satb1+ cells were preferentially concentrated in periventricular positions rather than distributed throughout the nucleus as in the turtle. This arrangement suggests that the medial portion of the aDVR (m-aDVR) represents a specialized cellular subgroup within the nucleus, distinguishing it from the lateral portion (l-aDVR), which contained a higher number of Ctip2+ cells (Fig. 5A’, B’, C’; Video S4). Likewise, the pDVR in the gecko contained Ctip2+ but not Satb1+ cells (Fig. 5D’, E’; Video S4), expanding, from the intermediate levels, throughout the VP to the most caudal regions (see both DVR subdivisions in Fig. 5E’), with the exception of the nucleus sphericus.

Taken together, these results highlight the conserved organization of the DVR as the main ventral pallial structure, suggesting the homology of this territory and its derivatives across both reptilian lineages (Fig. 5F). Nonetheless, in the gecko, potential specialized subdomains (m/l-aDVR) were observed that are absent in the turtle.

### Molecular profile and organization of the amygdala in reptiles

**Figure 6:**
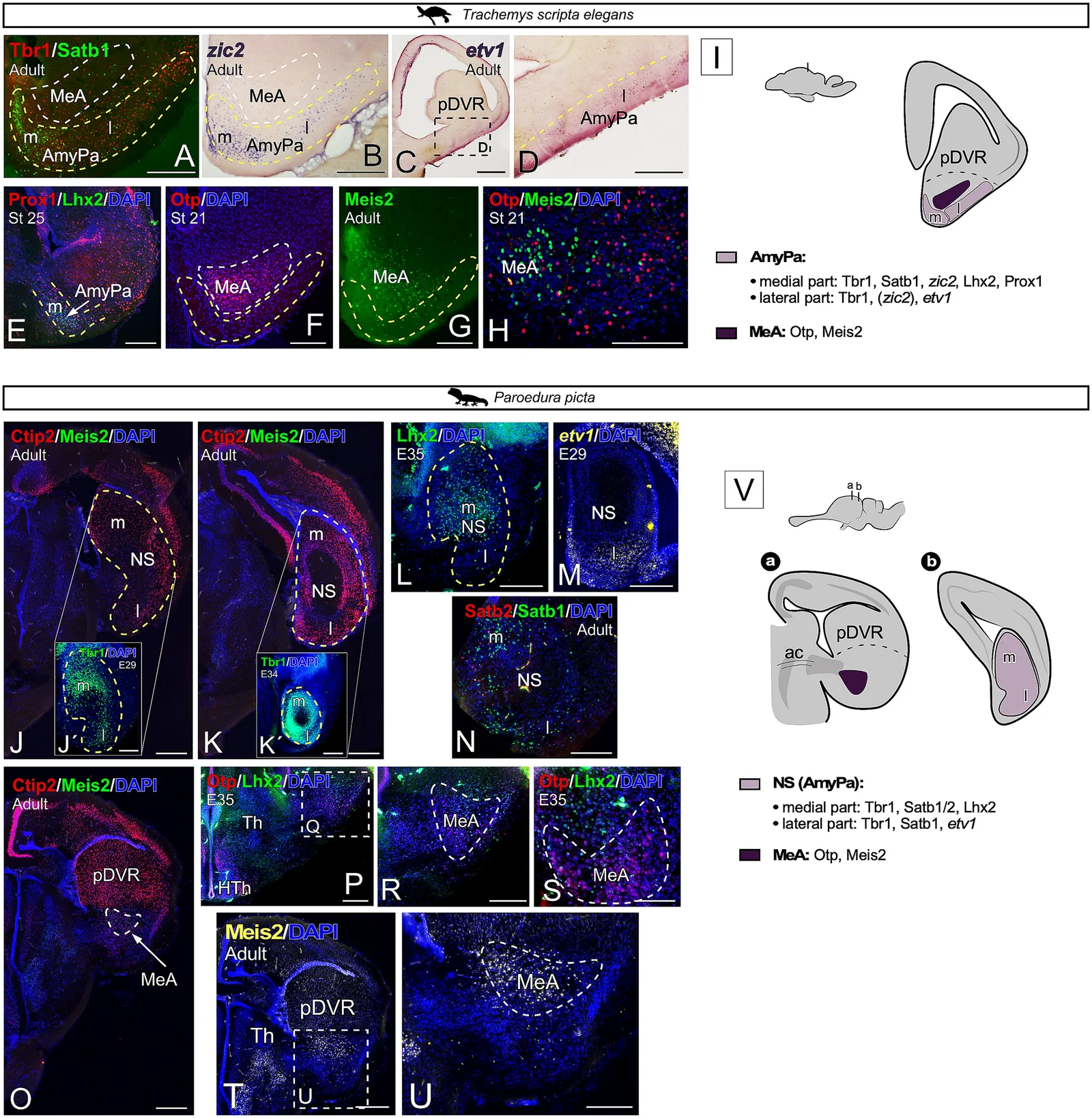
Molecular mapping of amygdalar subdivisions in turtle and gecko. Expression of Tbr1, *zic2, etv1*, Lhx2, Satb1, Otp, and Meis2 in amygdalar regions of *Trachemys scripta elegans* and *Paroedura picta*. The combination of these markers distinguishes amygdalar territories of pallial and medial identity. (A–H) In turtle, the panels show the pallial amygdala (AmyPa) and the medial amygdala (MeA). The AmyPa contains Tbr1^+^ cells and shows differential expression of Satb1, Zic2, Lhx2, Prox1, and etv1 in its medial and lateral subdomains. The MeA is identified by Otp and Meis2 expression and by low levels of Tbr1. (J–U) In gecko, the nucleus sphericus (NS) shows a molecular organization comparable to that of the turtle AmyPa. The medial subdomain of the NS expresses Satb1/Satb2 and Lhx2, whereas etv1 is preferentially detected in the lateral portion. The gecko MeA expresses Otp and Meis2 and is located rostrally to the NS. (I, V) Schematic summaries of amygdalar organization in turtle and gecko. The yellow outline delimits the AmyPa in turtle and the NS in gecko. The white outline delimits the MeA. Abbreviations: AmyPa, pallial amygdala; HTh, hypothalamus; l, lateral portion of the AmyPa or NS; m, medial portion of the AmyPa or NS; MeA, medial amygdala; NS, nucleus sphericus; pDVR, posterior portion of the DVR; Th, thalamus. Scale bars: A, B, D–G, J’, K’, L–M, R, U = 200 μm; C, J, K, O, P, T = 500 μm; H, S = 100 μm.

The amygdala is a fundamental integrative center for the processing of olfactory, social, and emotional information in vertebrates and is considered a structure of mixed origin, derived from pallial, subpallial, and even extratelencephalic territories (García-Moreno et al. 2010; Medina et al. 2023; Morales et al. 2025). Despite its functional and evolutionary importance, the molecular composition and embryonic origin of its subdivisions remain poorly characterized in non-avian reptiles. To address this gap, we examined the amygdaloid complex in *T. scripta elegans* and *P. picta* using a set of transcription factors selected for their conserved expression in other amniotes (Fig. 6).

In both species, the amygdaloid regions analyzed consisted of a set of nuclei organized into at least two major subpopulations, located in the caudal portion of the telencephalon, within a transitional zone between the pDVR and hypothalamic territories (Fig. 6C, J, K, O). The first domain, identified as the pallial amygdala in turtles and its homolog in the gecko, the nucleus sphericus (AmyPa and NS; yellow dashed lines in Fig. 6), was characterized by a substantial presence of Tbr1-positive cells (Fig. 6A, J’, K’) and exhibited a molecular marker combination consistent with that described for the mammalian pallial amygdala. Both the AmyPa and the NS were further subdivided into medial (m-) and lateral (l-) subunits. A higher cellular density was observed in the medial subdivision, whereas labeled cells in the lateral subdivision appeared more sparsely distributed. These regions displayed differential molecular expression patterns. In the m-AmyPa, Satb1, *zic2*, Lhx2, and Prox1 predominated (Fig. 6A, B, E). Comparably, the m-NS exhibited strong expression of Satb1/2 and Lhx2 (Fig. 6L, N). In contrast, *etv1* was more prominently expressed in the lateral portion of both structures (Fig. 6C, D, M).

The second domain, corresponded to the medial amygdala (MeA; white dashed lines in Fig. 6), which is considered predominantly of extrapallial origin. This domain was defined by robust Otp expression and low Tbr1 expression (Fig. 6F, P–S; compare Fig. 6A and F). Otp is a well-established marker of the MeA in vertebrates (Bardet et al. 2008; García-Moreno et al. 2010; Garcia-Calero et al. 2020; Medina et al. 2023; Morales et al. 2025). Additionally, Meis2 was detected in the MeA of both species (Fig. 6G, H, O, T, U). Notably, in the gecko, the MeA (Otp^+^, Meis2^+^) was positioned more rostrally than the AmyPa (Fig. 6O). In turtles, both amygdaloid domains were located within a similar caudal plane and exhibited a less prominent core–shell organization (compare Fig. 6A, B, F, G).

Together, these results highlight a remarkable conservation of the molecular architecture of the amygdala between the two reptile species, with domains that maintain equivalent topological positions and molecular identities (Fig. 6I, V).

### Three-dimensional reconstruction of pallial domains

To obtain a comprehensive 3D view of telencephalic organization, we employed light sheet fluorescence microscopy, which enables high-resolution volumetric imaging of cleared, intact brain tissue. Unlike traditional histological sectioning, this approach preserves anatomical continuity, allowing the reconstruction of complex spatial relationships between adjacent or overlapping pallial domains.

**Figure 7:**
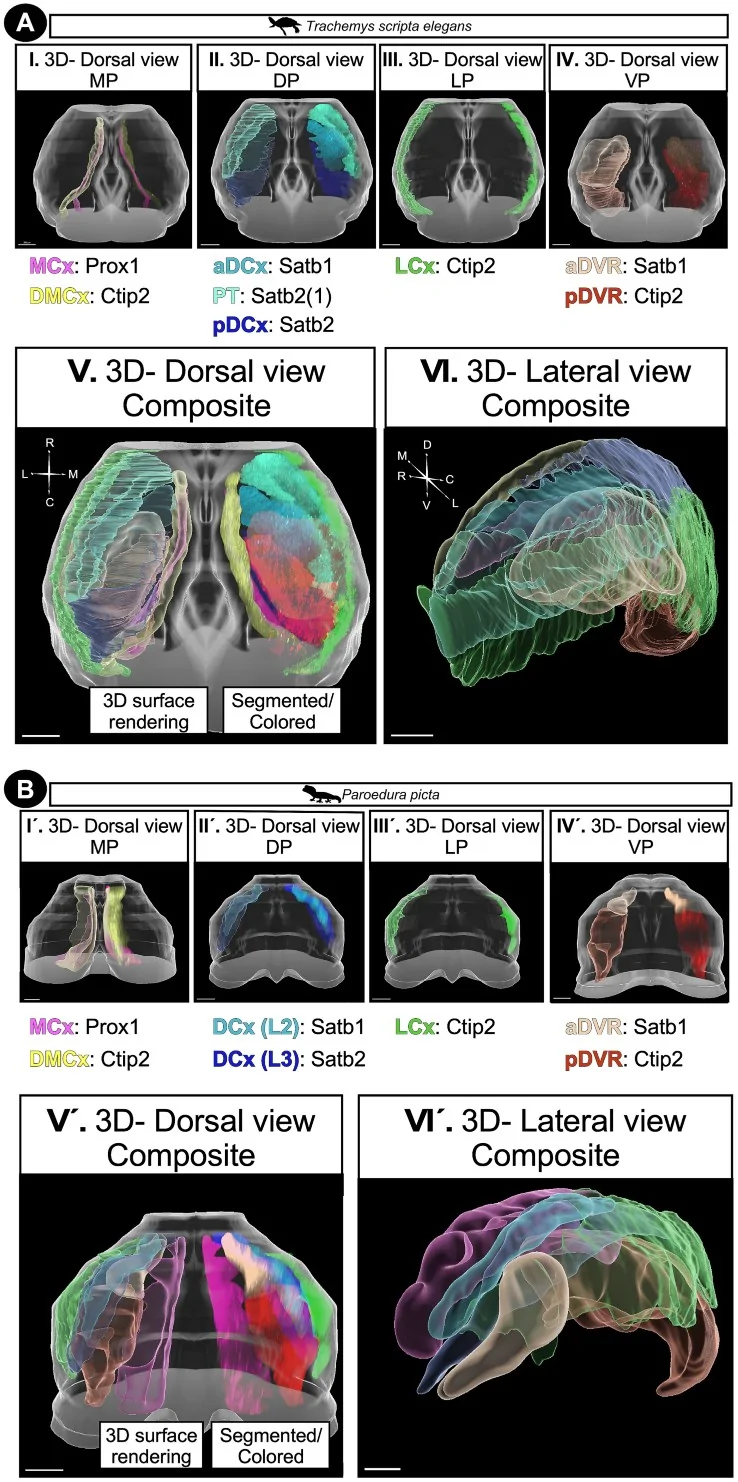
Three-dimensional reconstruction of pallial subdivisions in turtle and gecko. Volumetric reconstructions of the main pallial regions in *Trachemys scripta elegans* and *Paroedura picta* obtained by light-sheet fluorescence microscopy. Pallial domains were segmented based on Ctip2, Prox1, Satb1, and Satb2 expression. (A–I, B–I’) Reconstruction of medial pallial domains, Ctip2 and Prox1 delineate the medial cortex (MCx) and dorsomedial cortex (DMCx) in both species. (A–II, B–II’) Reconstruction of dorsal pallial domains. In turtle, the anterior dorsal cortex (aDCx), posterior dorsal cortex (pDCx), and pallial thickening (PT) are distinguished. In gecko, comparable populations are organized as DCx-L2 and DCx-L3. (A–III, B–III’) Reconstruction of the lateral cortex, the LCx was segmented mainly based on Ctip2 expression, owing to limitations in the 3D detection of other lateral pallial markers. (A–IV, B–IV’) Reconstruction of the DVR, combined Satb1 and Ctip2 expression distinguishes the anterior DVR (aDVR) and posterior DVR (pDVR) in both species. (A–V, B–V’) Integrated dorsal view of pallial subdivisions. In each panel, the left hemisphere shows the segmented domains, whereas the right hemisphere shows the corresponding 3D surface rendering. In V’ and VI’, the MCx and DMCx are shown together in pink. (A–VI, B–VI’) Lateral view of the 3D surface rendering, showing the spatial continuity of pallial domains. These reconstructions provide a 3D framework for comparing the spatial organization of molecularly defined pallial domains in turtle and gecko. Abbreviations: aDVR, anterior portion of the DVR; DCx, dorsal cortex; DMCx, dorsomedial cortex; DVR, dorsal ventricular ridge; LCx, lateral cortex; MCx, medial cortex; pDVR, posterior portion of the DVR; PT, pallial thickening. Scale bars: I–IV = 500 μm; II’, III’, V’ = 400 μm; I’, IV’, VI’ = 300 μm.

Applying this methodology to reptiles for the first time allowed us to visualize pallial territories within their full spatial context and identify interzonal boundaries that are often indistinguishable in conventional sections. We generated detailed 3D reconstructions of the main pallial domains along the anteroposterior axis in both adult and juvenile individuals of the two species (Fig. 7; Videos S1–S4). Volumes corresponding to the expression of representative pallial markers (Ctip2, Prox1, Satb1, and Satb2) were segmented to precisely delineate each structure.

In both species, Ctip2 and Prox1 expression defined the MCx (pink), whereas the combination of Ctip2/Prox1 with Satb1 helped delineate the DMCx (yellow), characterized by a Ctip2^−^/Prox1^−^/Satb1^+^ profile. Both cortical territories extended continuously from anterior to posterior levels (Fig. 7A–I, B–I’; Videos S1–S4). In the gecko, these cortices appeared more expanded dorsally (compare Fig. 7A–V and Fig. 7B–V’). In the complete gecko reconstruction (Fig. 7B–V’), the MCx and DMCx are represented together as a single medial cortical domain. This is because the marker combination used for this reconstruction (Satb1/Satb2/Ctip2) allowed the simultaneous delineation of most pallial territories, but did not permit an unambiguous distinction between the MCx and DMCx. By contrast, in the Prox1/Ctip2-based reconstruction (Fig. 7B–I’), these two medial pallial subdivisions could be distinguished and are therefore shown as separate territories. Satb1 and Satb2 expression delineated the most dorsal pallial portions, including the aDCx in the turtle and layer 2 of the DCx in the gecko, structures proposed here as homologous (Fig. 7A–II and Fig. 7B–II’; Videos S1–S4). The PT, identified as a prominent Satb2^+^ domain located beneath or adjacent to the Satb1^+^ aDCx region at anterior/intermediate levels, was evident only in turtle. Rather than implying that the turtle PT accounts for the laminar organization of the gecko dorsal cortex, our data indicate that Satb1^+^ and Satb2^+^ dorsal pallial populations are organized differently in the two species: they are distributed along the rostrocaudal axis in turtle, partly in relation to the PT, whereas in gecko comparable marker-defined populations are arranged as laminar subdivisions within the dorsal cortex. Satb2 further defined the pDCx in turtles and layer 3 of the DCx in geckos (Fig. 7A–II and Fig. 7B–II’). Due to immunodetection limits, the key marker for aLCx (Meis2) could not be visualized, and Satb1 signal in pLCx was insufficient for detailed surface reconstruction. Consequently, the LCx (green) was delineated by the Ctip2 expression (Fig. 7A–III, 7B–III’; Videos S1–S4). Adjacent to it, Satb1 and Ctip2 expression distinguished the two DVR subdivisions: aDVR (light brown) and pDVR (dark brown) (Fig. 7A–IV, Fig. 7B–IV’; Videos S1–S4). The aDVR appeared at more anterior levels in the gecko than in the turtle, where it occupied intermediate positions (compare Fig. 7–II, Fig. 7IV, and Fig. 7IV’; Videos S1–S4); in the latter, both DVR subdivisions overlapped medially, while in the gecko both remained largely independent (Fig. 7–IV’; compare with Ctip2/Satb1 expression in Videos S3–S4; see also Videos S1 and S2). The pDVR narrowed caudally in the gecko but remained thickened in the turtle.

Complete 3D reconstructions displayed all pallial domains in both hemispheres (Fig. 7A–VI, Fig. 7B–VI’) and as rotatable volumes in videos. These datasets reveal the spatial continuity among pallial regions, providing an integrated anatomical framework that complements and extends the molecular mapping.

### Radial glia orientation in the turtle and gecko pallium

In mammals, radial glia function not only as neural progenitors and guides for neuronal migration but also exhibit region-specific architectures that correlate with cortical arealization and laminar complexity (Kriegstein and Alvarez-Buylla 2009). Similarly, in reptiles and other non-mammalian vertebrates, radial glial fibers display spatially distinct trajectories that align with the topography of pallial subdivisions, even in the absence of overt lamination (Nomura et al. 2013a). Thus, the orientation and regional organization of radial glial scaffolds have been used as anatomical landmarks of the underlying telencephalic histogenetic domains (Garcia-Calero and Puelles 2020).

**Figure 8:**
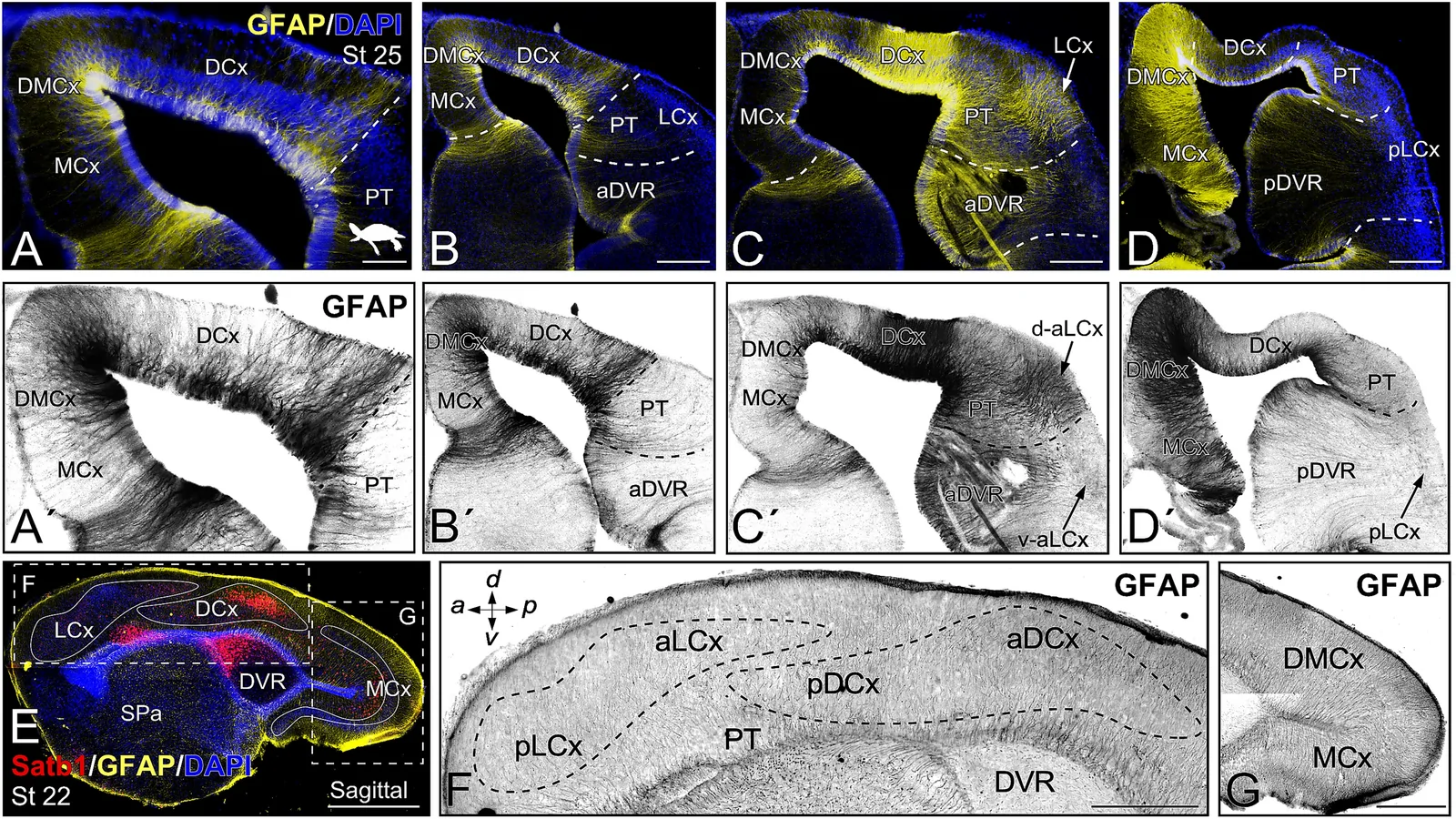
Radial glia trajectories in pallial subdivisions of turtle. GFAP^+^ radial glial cells in *Trachemys scripta elegans* visualized by immunohistochemistry. The orientation and density of GFAP^+^ radial glial fibers reveal regional differences in radial glial organization across pallial territories. These patterns were interpreted as complementary anatomical information relative to molecularly defined pallial domains, rather than as independent markers of sharp areal borders. (A–D’) Serial coronal sections of the pallium at an advanced developmental stage (St25). GFAP^+^ fibers extend from the ventricular surface toward the mantle and show region-specific orientations across medial, dorsal, lateral, and ventral pallial domains. (E–G) Sagittal sections stained for GFAP alone (F, G) or combined with Satb1 (E), used to relate radial glial organization to specific pallial domains along the anteroposterior axis. White and black dashed lines indicate boundaries between pallial domains. Abbreviations: aDCx, anterior portion of the dorsal cortex; aDVR, anterior portion of the DVR; aLCx, anterior portion of the LCx; d-aLCx, dorsal portion of the aLCx; DCx, dorsal cortex; DMCx, dorsomedial cortex; DVR, dorsal ventricular ridge; LCx, lateral cortex; MCx, medial cortex; pDCx, posterior portion of the dorsal cortex; pDVR, posterior portion of the DVR; pLCx, posterior portion of the LCx; PT, pallial thickening; v-aLCx, ventral portion of the aLCx. Scale bars: A–D’, F, G = 200 μm; E = 500 μm.

Based on this evidence, we employed GFAP immunostaining in *T. scripta elegans* at late developmental stages to examine whether region-specific patterns of radial glial orientation support or refine the anatomical demarcation of pallial territories previously identified through molecular markers (Fig. 8). The analysis was limited to developing turtle, as GFAP expression in the gecko telencephalon has already been thoroughly characterized in previous studies (Lazzari and Franceschini 2005; Lõrincz and Kálmán 2020). Radial glia was examined in coronal sections at stage 25 (Fig. 8A–D’) and in sagittal sections, combined with Satb1, at stage 22, which allowed precise delineation of distinct domains (Fig. 8E–G). Radial glial fibers extended continuously from the ventricular surface to the mantle across the entire pallium, although their orientation varied markedly depending on the region.

In the MCx and DCx, GFAP^+^ glial fibers exhibited a parallel arrangement along the ventricular-pial axis (Fig. 8A–D’). However, GFAP labeling did not define sharp areal borders by itself. Instead, fiber density and organization varied across pallial territories and along the anteroposterior axis. In particular, the dense GFAP labeling observed within the DCx and DMCx in coronal sections suggests regional differences, or possible gradients, in radial glial organization rather than discrete boundaries. Accordingly, we interpret GFAP as complementary anatomical information that supports, but does not independently define, the pallial domains established by transcription factor expression.

In the DP, changes in GFAP^+^ fiber density and orientation were broadly consistent with the molecularly defined topography of the DCx and adjacent PT. Whereas the DCx showed a mainly radial glial scaffold, the adjacent PT contained GFAP^+^ fibers with a more oblique trajectory, converging toward the ventricular apex and forming a dense organized arrangement (Fig. 8B–B’, C–C’). Similarly, the aLCx showed a curved orientation of GFAP^+^ fibers, with a prominent arc-shaped trajectory in its dorsal subregion (Fig. 8C–C’), continuous with the adjacent domain associated with the PT. By contrast, in the vLCx, GFAP^+^ fibers crossing the aDVR did not appear to extend into this region, resulting in comparatively weaker labeling (Fig. 8C–C’). These differences in radial glial organization are consistent with the proposed dorsoventral molecular subdivision of the LP and may reflect distinct developmental trajectories among these territories. The DVR exhibited densely packed GFAP+ fibers extending from the ventricular zone toward the surface, with a particularly prominent accumulation in its anterior portion (Fig. 8C–C’), highlighting its identity as a functionally distinct domain from its more caudal regions, pDVR (Fig. 8D–D’). In sagittal sections (Fig. 8E–G), GFAP labeling was weaker than in coronal material and did not by itself allow precise delineation of pallial subdivisions. For this reason, sagittal GFAP staining was interpreted in combination with Satb1 (Fig. 8E), which provided a more robust molecular reference for assigning the corresponding domains along the anteroposterior axis.

Taken together, both the spatial organization and regional abundance of radial glia in *Trachemys* closely mirror the boundaries previously established at the molecular level, supporting the hypothesis that this glial architecture actively contributes to the topological compartmentalization of the reptilian pallium during development.

### Identification of progenitor types in the turtle pallium

We have observed qualitative differences between the pallial regions of both species. These disparities likely reflect differences in the underlying developmental programs, particularly in the generation, specification, and amplification of distinct progenitor types. In this regard, previous studies have described differences between chelonians and lizards, such as the absence of basal radial glial cells (bRGCs) in geckos, which appear to be present, albeit sparsely, in turtles (Nomura et al. 2013a; Nomura et al. 2016).

**Figure 9:**
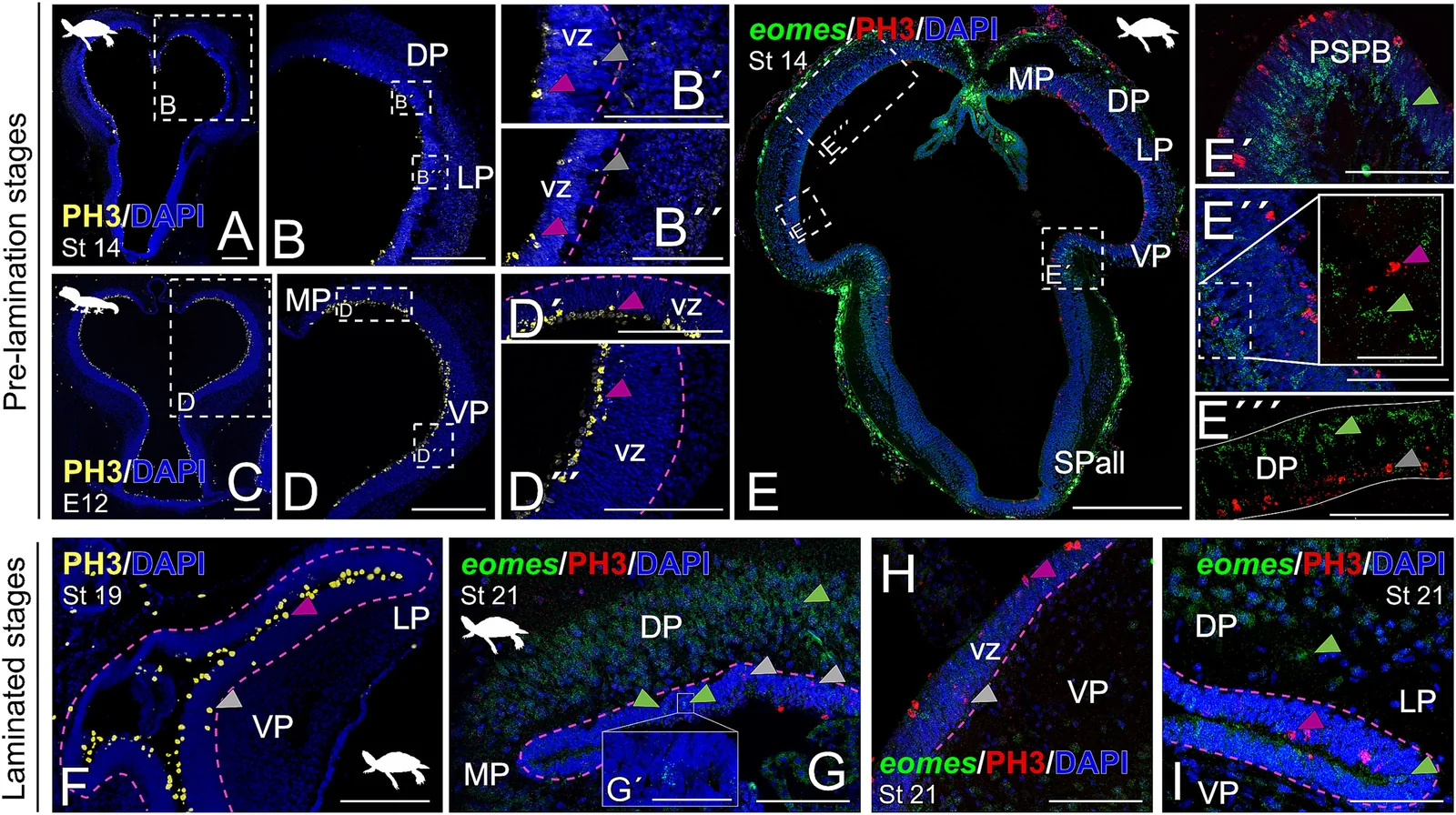
Identification of progenitor types in the pallium of turtle and gecko. Analysis of PH3^+^ mitotic cells and *eomes*^+^/Tbr2^+^ cells in the pallium of *Trachemys scripta elegans* and *Paroedura picta*. The panels compare the distribution of ventricular and abventricular mitoses at prelaminar and laminated stages. (A, B) In turtle, PH3 labels mitotic cells both at the ventricular surface and at abventricular positions. Ventricular mitoses correspond to apical progenitors, whereas abventricular mitoses are consistent with basal progenitors. (B’–B”) Higher-magnification views of ventricular and abventricular mitoses in the turtle pallium. (C, D, D’–D”) In gecko, PH3^+^ cells are located mainly at the ventricular surface, with no clear evidence of abventricular mitoses at the stages analyzed. In turtle, *eomes*^+^/Tbr2^+^ cells do not show co-expression with PH3 either before lamination (E–E”’) or at laminated stages (F–I), indicating that they do not correspond to mitotically active intermediate progenitors detectable with this approach. Magenta dashed lines delimit the ventricular zone. Magenta arrowheads indicate ventricular/apical mitoses. Gray arrowheads indicate abventricular/basal mitoses. Green arrowheads indicate postmitotic neurons. Abbreviations: DP, dorsal pallium; LP, lateral pallium; MP, medial pallium; PSPB, pallial-subpallial boundary; SPall, subpallium; VP, ventral pallium; VZ, ventricular zone. Scale bars: A–D, E, F = 500 μm; B’–D”, E’–E”, G–I = 200 μm; G’ = 50 μm.

To further investigate these observations and relate them to the qualitative differences described in this study between the pallial regions of both species, we analyzed the presence of proliferative progenitor cells in the pallium by assessing mitotic activity through immunolabeling for phosphorylated histone H3 (PH3) (Fig. 9). This analysis was conducted at developmental stages prior to cortical lamination in both reptiles (stage 14 in turtle, Fig. 9A–B” and stage E12 in gecko, Fig. 9C–D”). At these stages, mitotic cells were predominantly located at the luminal surface of the germinative ventricular zone (VZ; outlined with magenta dashed lines), a characteristic feature of apical radial glial cells (aRGCs; magenta arrowheads in Fig. 9B’, B”, D’, D”). It was exclusively in the turtle that a minor population was observed to display abventricular mitoses in proximity to the mantle zone. This finding suggests the presence of basal progenitors (BPs; gray arrowheads in Fig. 9B’, B”).

To determine whether these abventricular mitoses persisted once lamination was under way, PH3 immunolabeling was analyzed in turtles at a later stage, when the cortical layers were beginning to form (stage 19; Fig. 9F). These mitotic cells remained scarce at this stage and were restricted to the VP (gray arrowheads in Fig. 9F). It is worth noting that PH3-based mitotic activity was not evaluated in geckos at comparable or later stages, as no abventricular mitoses had been detected earlier in development. It is worth noting that PH3-based mitotic activity was not evaluated in geckos at post-lamination stages, as abventricular mitoses were not detected at earlier stages.

There is ongoing controversy regarding the presence of intermediate progenitor cells (IPCs) in the pallium of non-mammalian vertebrates. The absence of IPC in sauropsids has been described (Nomura et al. 2013a; Rueda-Alaña et al. 2025); however, recent findings have reported their presence in certain anamniotes, suggesting an earlier evolutionary origin than previously assumed (Deryckere et al. 2025; Quintana-Urzainqui et al. 2025). In this study, we investigated whether the described BPs in the turtle pallium expressed Eomesodermin (Eomes/Tbr2), the canonical marker of IPCs in mammals. We analyzed the co-expression of *eomes* and the mitotic marker PH3 at a pre-lamination embryonic stage (stage 14; Fig. 9E–E”’). *Eomes* expression was detected both in the mantle and ventricular zones across all pallial regions (MP, DP, LP and VP in Fig. 9E). However, no mitotically active *eomes^+^* cells were identified in any of these regions, including the pallial-subpallial boundary (Fig. 9E’), the VP (Fig. 9E”), and the DP (Fig. 9E”’). Similarly, at stage 21, after cortical layer formation, no proliferative *eomes^+^* cells were observed (green arrowheads in Fig. 9G–I). It is important to note that the mitotic marker PH3 specifically identifies cells in the M phase of the cell cycle; therefore, the presence of *eomes* + cells in other phases of the cell cycle cannot be ruled out. Thus, the results suggested that the *eomes*-expressing cells corresponded to postmitotic *eomes^+^* neurons, but not to IPCs. This methodological limitation constrains our conclusions, as it does not allow us to rule out the presence of *eomes^+^* cells in other phases of the cell cycle that are not detectable by PH3, such as G1, S, or G2.

These findings support the hypothesis that, as in birds, IPCs may be absent in sauropsids, and that the scarce BPs detected in the turtle pallium may belong to the bRGC population (Nomura et al. 2013a; Nomura et al. 2016).

### Evolutionary synthesis of pallial organization in amniotes

**Figure 10:**
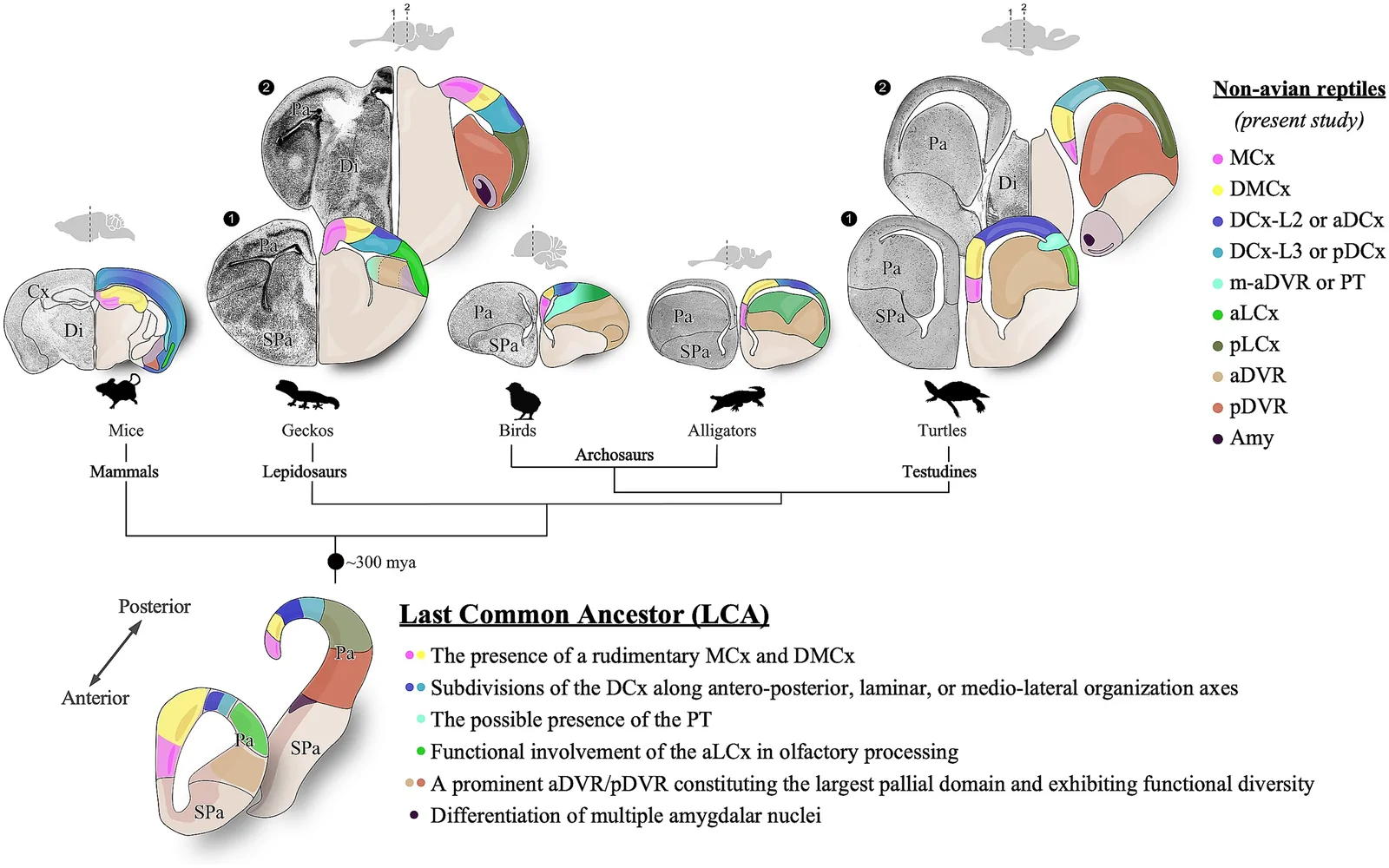
Representative scheme of pallial subdivisions in amniotes. Phylogenetic diagram illustrating coronal sections of the telencephalon in amniotes. In all cases, the diagram shows Nissl staining in the left hemisphere and a schematic representation of pallial subdivisions in each model in the right hemisphere, with special emphasis on the subdivisions described in the present study in reptiles (gecko and turtle). In these species, two histological sections ordered along the rostrocaudal axis are depicted in order to illustrate both anterior and posterior subunits. The scheme highlights that the last common ancestor of amniotes likely already possessed all pallial subdivisions, which subsequently evolved in a lineage-specific manner. The figure has been modified from (Jiménez et al. 2026) in accordance with the results of the present study. Abbreviations: aDCx, anterior part of DCx; aDVR, anterior part of DVR; aLCx, anterior part of LCx; Amy, amygdala; Cx, cortex; DCx, dorsal cortex; DCx-L2/3, layer 2 or 3 of the DCx; Di, diencephalon; DMCx, dorsomedial cortex; m-aDVR, medial part of the aDVR; MCx, medial cortex; mya, million years ago; Pa, pallium; pDCx, posterior part of the DCx; pDVR, posterior part of the DVR; pLCx, posterior part of the LCx; PT, pallial thickening; SPa, subpallium.

To integrate the genoarchitectonic observations obtained in turtle and gecko with previously described pallial organizations across amniotes, we generated a comparative schematic reconstruction summarizing the main pallial domains and their inferred evolutionary relationships (Fig. 10).

This integrative model highlights a set of conserved pallial territories that were likely already present in the last common ancestor of amniotes, including the medial pallium (MCx), dorsomedial pallium (DMCx), lateral pallium (LCx), and ventral pallial derivatives such as the DVR. In addition, our comparative analysis suggests that the DP exhibits lineage-specific structural rearrangements across reptilian lineages, including the rostrocaudal subdivision observed in turtles and the laminar organization detected in geckos.

Together, this evolutionary framework illustrates how conserved neuronal transcriptional programs may have been spatially reorganized during amniote diversification, giving rise to the distinct pallial architectures observed in extant lineages.

## Discussion

### Genoarchitectonic conservation of the medial pallium in amniotes

Our findings reveal a pronounced conservation of the genoarchitectonic organization of the MP in turtle and gecko. The expression of Prospero Homeobox 1 (Prox1), a key marker associated with the maturation of postmitotic granule cells in the mammalian dentate gyrus (DG) (Lavado et al. 2010) is replicated in the V-shaped hippocampal region of birds (Atoji et al. 2016) and in the MCx of crocodilians (Briscoe and Ragsdale 2018b). Our identification of Prox1 expression in the MCx of chelonians and gecko substantially broadens the phylogenetic range of reptiles exhibiting this molecular program, consistent with previous reports in *pogona* (Tosches et al. 2018). Complementarily, the presence of BCL11 Transcription Factor B (Ctip2^+^) in the MCx suggests parallels with granule-cell differentiation in the DG (Simon et al. 2016). Likewise, the expression of ETS Variant Transcription Factor 1 (*etv1*) consistently marks the DMCx in both species, supporting its interpretation as a CA-related medial pallial domain. This *etv1*^+^ DMCx lies adjacent to the Prox1^+^/Ctip2^+^ MCx, thereby preserving a topological relationship broadly comparable to the DG–CA organization of the mammalian hippocampal formation and consistent with proposed homologous domains in birds (Gupta et al. 2012) and crocodilians (Briscoe and Ragsdale 2018b). Taken together, these data support the hypothesis that the MP represents the ancestral precursor of the mammalian hippocampus and its para−/hippocampal extensions. This interpretation aligns with recent transcriptomic evidence in birds, where MP neurons retain more conserved gene-expression profiles than those of other pallial domains (Zaremba et al. 2025). Moreover, recent large-scale comparative studies extend this conservation even further, identifying a homologous medial pallial/hippocampal domain throughout tetrapods, including amphibians, based on shared developmental trajectories, gene regulatory programs, and circuit-level organization (Jiménez and Moreno 2022; Woych et al. 2022). Additional support arises from the long-standing conservation of core hippocampal functions, such as spatial navigation and memory consolidation (Rodríguez et al. 2002; Striedter 2016), as well as from its remarkably stable hodological organization across amniotes (Ulinski 1975; Bruce and Butler 1984a; Bruce and Butler 1984b; Ulinski 1990; Hoogland et al. 1998).

Despite this deep cellular conservation, we also observed substantial structural variability across lineages. Examples include the dorsalization of the MCx/DMCx in lepidosaurs, the looser trilaminar organization in turtle (present study), and the non-laminar arrangement reported in birds (Striedter 2016). These differences indicate that, although the MP constitutes a cellularly and genetically stable domain, its cellular architecture has been repeatedly reshaped during amniote evolution.

### Lineage-specific remodeling of the dorsal cortex

The DCx exhibits a strong molecular correspondence across non-avian reptiles, yet with clear lineage-specific structural divergence. The anterior DCx (aDCx) of turtle, characterized primarily by Satb1^+^/Ctip2^+^ expression and absence of *etv1*, shows molecular similarities with DCx-L2 in gecko, as well as with the medial dorsal cortex (DCm) of crocodilians (Briscoe and Ragsdale 2018b) and *Pogona vitticeps* (Tosches et al. 2018). At anterior turtle levels, this domain must be interpreted in relation to the neighboring Satb2^+^ pallial thickening (PT), which occupies the much of the pallium rostrally and progressively shifts toward a deeper central position. Thus, Satb2^+^ cells observed beneath or adjacent to the Satb1^+^ aDCx band should not be assigned exclusively to the aDCx, as they include a substantial PT-associated component. Conversely, the posterior DCx (pDCx), defined by prominent Satb2 expression and reduced Satb1 levels, corresponds molecularly to gecko DCx-L3 and to the lateral dorsal cortex (DCl) of crocodilians (Briscoe and Ragsdale 2018b), reflecting a conserved dorsal pallial subdomain across sauropsids.

The rostrocaudal gradient observed in turtle, and its reorganization into a laminar arrangement in gecko, indicate that dorsal pallial diversification in non-avian reptiles primarily reflects spatial redeployment of ancestral transcriptional cell types rather than the acquisition of novel gene regulatory programs (Butler et al. 2011; Briscoe and Ragsdale 2018a; Tosches 2021; Zaremba et al. 2025). These differences highlight an evolutionary plasticity that is primarily structural, reflecting the spatial and laminar reorganization of conserved neuronal cell types rather than changes in neuronal differentiation programs, which appear to remain remarkably stable and shared across reptilian lineages.

This subdivision is most parsimoniously interpreted as reflecting functional and molecular specialization within a single pallial domain, rather than distinct embryological or topographical identities. Whether the laminar arrangement of the gecko DCx (DCx-L2/DCx-L3) arises from neurons of distinct neuroepithelial origin that migrate to form separate layers, or from a redeployment of a common progenitor pool, cannot be resolved with our data: addressing this would require radial-glia/lineage information that is not available in the published record for these species and that lies beyond the resolution of our GFAP labeling. We therefore present the single-domain interpretation as the most parsimonious reading of the molecular data, while noting that a dual-origin scenario for L2 and L3 remains to be tested directly. This finding is consistent with previous studies that have suggested the existence of functional or topographical subdivisions within the turtle DCx (Dugas-Ford et al. 2012; Tosches et al. 2018). Functional parallels with mammals, where Satb1 marks thalamorecipient neurons, Satb2 denotes intratelencephalic projections, and Etv1 identifies corticofugal pathways (Briscoe and Ragsdale 2018a; Rueda-Alaña et al. 2025), suggest that these core regulatory architectures were already present in the last common ancestor of amniotes. Although neocortical lamination is not directly homologous to reptilian DCx organization, conserved transcriptomic and functional gradients support the existence of a shared dorsal transcriptional module that has undergone lineage-dependent spatial and regulatory remodeling throughout amniote evolution.

In this regard, it has been reported that the gecko’s DCx shares several efferent characteristics with the ventral subiculum of mammals, including projections to the septum, the hypothalamus, and the nucleus accumbens (Hoogland and Vermeulen-Vanderzee 1989). Although connectivity alone does not establish homology, these hodological data may help refine hypotheses based on molecular evidence. That said, similarities in connectivity may also reflect convergent evolution rather than a common origin. Therefore, although these observations suggest that the DCx may share some circuit characteristics with the subicular regions, further analyses, including the use of subicular markers, as well as a more detailed characterization of its connectivity, will be necessary to evaluate this hypothesis.

Finally, our study revealed the presence of the PT exclusively in turtles. In the gecko, we did not identify any comparable nuclear organization, even at the most rostral levels, where the PT has previously been described in lepidosaurs (Desfilis et al. 2018). The presence of this prominent structure in turtles, further characterized by a specific combination of gene expression, may reflect the conservation of an ancestral pallial organization within the chelonian lineage. From a functional perspective, the PT has been shown to receive substantial sensory input, particularly visual, originating from the thalamus, specifically the dorsal lateral geniculate complex (Heller and Ulinski 1987). This connectivity pattern resembles that described for the dorsal cortex (DCx) in lepidosaurs, which also constitutes a major target of visual thalamic afferents (Desfilis et al. 1998; Jiménez et al. 2026). Moreover, in reptiles this region has been proposed to show functional correspondences with pallial territories such as the aDVR, or even the claustrum, particularly in relation to sensory integration and the regulation of brain states, including sleep control [(Norimoto et al. 2020), *see below*]. In this regard, Norimoto et al. (2020) identified several transcriptomic clusters located in the turtle PT that show similarities with the amDVR clusters of *Pogona*. Thus, the turtle PT and the lepidosaurian amDVR may share a related transcriptomic identity, despite their clearly distinct anatomical organization. This interpretation is consistent with the absence, in our gecko material, of a nuclear territory equivalent to the turtle PT. Although a PT has been described in other gecko species based on Nissl cytoarchitecture (Puelles et al. 2017), our molecular data suggest that the region occupying a comparable topological position in *P. picta* corresponds to DCx-L2, characterized by Satb1 expression, rather than to an independent PT-like domain. In this context, molecular markers help refine the anatomical interpretation of this region and distinguish between topological similarity and true molecular or genoarchitectonic equivalence.

Taken together, these findings suggest that the differences observed between lineages do not necessarily reflect the emergence or loss of entire circuits, but rather divergent evolutionary strategies in pallial organization.

### Transcriptional and hodological continuity in the lateral cortex

The expression of *reelin* and Meis2 in the reptilian LCx and in the mammalian piriform cortex, along its olfactory input through the lateral olfactory tract (Ulinski and Rainey 1980; Butler 1994; Ubeda-Bañon et al. 2011), supports the existence of a conserved transcriptional program within the olfactory system of amniotes (Goffinet et al. 1999; Stranahan et al. 2011; Agoston et al. 2014; Tosches et al. 2018). Similarly it has been observed that the anterior LCx is receiving the densest olfactory input, and, in snakes, it is enriched in *bowl cells* (Ulinski 1990), which are likely homologous to mammalian semilunar cells of the piriform cortex; both cell types express *reelin*  ^+^ (Goffinet et al. 1999; Diodato et al. 2016; Klingler 2017). This is reinforced by the existence of the internal subdivision of the LCx documented across snakes, geckos and lacertids (Ulinski and Rainey 1980; Hoogland and Vermeulen-Vanderzee 1995; Guirado and Dávila 2002). Moreover, the described projection from the LCx to the MCx (Ulinski and Rainey 1980; Desan 1984) parallels the mammalian entorhinal–DG/CA3 pathway (Witter 2007; Leitner et al. 2016), further supporting the view that the anterior LCx represents an ancestral component of an olfactory–entorhinal–hippocampal circuit (Tosches 2021). Consistent with this, an injection of neurobiotin into the ventral portion of the turtle OB labeled the fibers reaching the LCx, providing direct confirmation of the existence of a bulbar projection to the lateral cortex in our material. Furthermore, given that it has been proposed that part of the turtle dorsal OB corresponds to the accessory OB (Franceschini et al., 1996), it could be suggested that the vomeronasal projections were not reached in our specimens.

Conversely, the restriction of Special AT-rich sequence-binding protein 1 (Satb1) expression to the posterior LCx suggests a distinct molecular specialization oriented toward thalamo–pallial integration. Indeed, it aligns with connectivity analyses conducted in some reptilian species, showing that the dorsolateral anterior thalamic nucleus projects bilaterally, and more densely, to posterior LCx sectors (Guirado and Dávila 2002). In some lineages, the resulting anteroposterior polarity thus reflects the coexistence of two ancient functional modules within the LCx: an anterior olfactory module and a posterior thalamorecipient one.

### Internal regionalization and functional divergence within the DVR

The DVR of both turtle and gecko exhibits a non-laminar architecture defined by complementary expression profiles of Satb1 and Ctip2. The anterior portion (aDVR) co-expresses both markers, whereas the posterior DVR (pDVR) is exclusively Ctip2^+^. However, only geckos display a further subdivision of the aDVR. In gecko, the subdivision into medial and lateral aDVR (m-aDVR/l-aDVR), with abundant periventricular Satb1^+^ cells in the medial sector (Rueda-Alaña et al. 2025), parallels transcriptomic diversities described in other lepidosaurs (Norimoto et al. 2020; Schede et al. 2021). The transcriptomic and functional similarities described between the m-aDVR, the turtle PT, and the mammalian claustrum (Tosches et al. 2018; Norimoto et al. 2020) suggest an evolutionary link grounded in shared neuronal types, likely long-range projection neurons with multimodal integrative capacities and potential auditory inputs from the medial thalamic nucleus (Foster and Hall 1978; Bruce and Butler 1984a; Bruce and Butler 1984b; Martínez-García and Lanuza 2009). This functional equivalence is preserved in crocodilians, where the m-aDVR also receives auditory projections (Pritz 1974), but it is absent in its transcriptomic homolog in birds, the mesopallium (Zaremba et al. 2025). Thus, the m-aDVR reflects a conservation of neuronal cell types, but not a complete preservation of sensory circuits across all amniotes.

By contrast, the l-aDVR of the gecko and the aDVR of the turtle, both Satb1^+^/Ctip2^+^, correspond to a molecular profile that, in birds, characterizes thalamorecipient neurons involved in visual and somatosensory processing (Martínez-García and Lanuza 2009; Briscoe et al. 2018; Rueda-Alaña et al. 2025), a functionality also reported in crocodilians and other non-avian reptiles (Pritz 1975; Martínez-García and Lanuza 2009). This thalamorecipient, sensory-recipient identity matches a defining feature of the mammalian basolateral complex, whose lateral nucleus (LA) is the principal entry point of sensory, including thalamic, information into the amygdala. The l-aDVR is therefore best compared with the LA, a correspondence supported both by connectivity (Bruce and Neary 1995) and by single-cell transcriptomics, in which aDVR glutamatergic neurons correlate specifically with the lateral amygdalar nucleus (Tosches et al. 2018). Unlike the m-aDVR, the l-aDVR may represent a visual and somatosensory integration module conserved across amniotes.

The pDVR’s exclusively Ctip2^+^ identity sets it apart from the aDVR. Ctip2 is associated with long-range glutamatergic projection neurons (Harb et al. 2016) and consistent with this the reptilian pDVR exhibits descending hypothalamic and striatal connections reminiscent of the basal (output) nuclei of the mammalian basolateral complex and of the cortical amygdala (Lanuza et al. 1998; Martínez-García et al. 2002). These efferents align the pDVR with the basal/output tier of the complex rather than with its sensory-recipient LA, which instead corresponds to the aDVR (Tosches et al. 2018). The basolateral correspondence of the reptilian DVR is thus distributed across both subdivisions: a sensory-recipient anterior tier (l-aDVR corresponds to LA) and a basal/output posterior tier (pDVR corresponds to basal and cortical nuclei). This interpretation is further supported at the functional level by lesion studies in lizards and crocodilians, which document disruptions in tonic immobility and escape behaviors analogous to those observed after BLA damage in mammals (Keating et al. 1970; Davies et al. 2002). Collectively, these findings indicate that the pDVR represents a phylogenetically stable node involved in descending sensory processing and evolutionarily conserved emotional circuits.

### Amygdalar pallial domains and sensory-driven diversification

Another region that exhibits marked morphological differences between species, despite sharing a broadly similar molecular repertoire, is the pallial amygdala. Following the analysis of the molecular markers and the neuroanatomic topography, our observations in these models showed that in turtles, the pallial amygdaloid region forms a continuous superficial layer to the pDVR, while in geckos it adopts a particular anatomical organization, defined as a spherical nucleus (NS).

Specifically, in the turtle, it was composed of two subunits, medial and lateral, both of which expressed Satb1, *zic2*, Lhx2, and *etv1*, characteristic of the mammalian pallial amygdala (Remedios et al. 2004; Garcia-Calero et al. 2020; Garcia-Calero et al. 2021; Fernández et al. 2025). A notable observation was the presence of Lhx2 expression in the medial region and etv1 in the lateral region. Lhx2 has been implicated in the development of the lateral olfactory tract in mammals (Saha et al. 2007), which could explain its presence in the reptilian AmyPa and its association with olfactory processing. Thus, it suggests that the medial region of the AmyPa may correspond to or share characteristics with the nucleus of the lateral olfactory tract in mammals, while the lateral portions may be related to other pallial amygdala regions, such as the cortical amygdala. Similarly, the nucleus sphericus of the gecko has been previously described as a component of the amygdaloid complex (Smeets et al. 1986;Lohman et al. 1988; Martínez-García et al. 2002). In agreement with these classical descriptions, our results show that this structure exhibits a molecular signature characteristic of pallial amygdaloid territories, including the expression of Satb1, *zic2*, Lhx2, and *etv1*. Based on this evidence, we propose that the nucleus sphericus corresponds to the vomeronasal-recipient cortical component of the gecko pallial amygdala, rather than to the pallial amygdala in its entirety. Following Martínez-García et al. (2002), the squamate pallial amygdala is a continuum that also comprises a secondary-olfactory cortical moiety (ventral anterior amygdala) and a deep, basolateral-like moiety (posterior DVR and lateral amygdala, together with the lateropallial dorsolateral amygdala); the nucleus sphericus represents only its vomeronasal cortical core. In both species, the MeA was located adjacent to these outer regions and was identified by Otp expression, a hypothalamic-derived marker conserved in all the vertebrates analyzed (Lozano et al. 2025), together with Meis2, which is associated with inhibitory populations typical of non-pallial amygdalar nuclei (García-Moreno et al. 2010; Metwalli et al. 2022; Hochgerner et al. 2023).

However, the interspecific differences become more pronounced when connectivity is considered. In turtles, the pallial amygdala receives direct projections from the main olfactory bulb (MOB), whereas the accessory olfactory bulb (AOB) is comparatively reduced and provides only limited vomeronasal input (Chkheidze and Belekhova 2005; Abdali et al. 2020). As a result, this region forms a predominantly olfactory, laminar domain reminiscent of the mammalian nucleus of the lateral olfactory tract layer 1–anterior cortical amygdaloid nucleus–anterior amygdaloid area (nLOT1–ACo–AA) complex. In geckos, by contrast, the pronounced vomeronasal specialization characteristic of squamates, facilitated by the bifid tongue and tongue-flicking behavior, results in a highly developed AOB (Schwenk 2008). Consequently, in rostral sections the MeA appears first, while more caudally the pallial amygdala expands around it to form the nucleus sphericus. MOB afferents remain superficial, whereas AOB projections penetrate deeply into the central core (Lohman et al. 1988; Martínez-García et al. 2002). The emerging structure is therefore hybrid: an outer layer with olfactory characteristics (nLOT1/ACo/AA-like) and a central domain resembling vomeronasal regions (posteromedial cortical amygdaloid nucleus, PMCo-like).

The present analysis of the amygdaloid territory focuses on its superficial, chemosensory moiety (the olfactory and vomeronasal cortical amygdala) and on the medial amygdala, because the marker panel used here (Tbr1, *zic2*, Satb1/2, Lhx2, Prox1, *etv1*, Otp, and Meis2) was selected to resolve these layered/cortical and medial identities. The deep, basolateral-like nuclei of the pallial amygdala were therefore not delineated as individual territories in this section. In squamates and chelonians these deep components largely correspond to the DVR, whose cell-type identity we interpret above within the claustroamygdalar framework (Bruce and Neary 1995). Consistent with this, the *etv1*^+^ lateral subdomain of the AmyPa/NS described here already carries a basolateral-type molecular signature (Tosches et al. 2018), indicating that a basolateral-like identity is captured within the analyzed territory even though its full nuclear parcellation lies beyond the present marker set.

Our findings indicate that, although turtles and geckos share a common pallial genetic cell type toolkit, each lineage reorganizes these modules according to their sensory demands: a simpler, laminar, olfaction-dominated pallial amygdala in turtles versus a nuclearized, strongly vomeronasal one in geckos. It should be noted that this study focused on pallial amygdaloid structures; subpallial-derived components of the amygdala were not analyzed.

### Radial glia as a low-resolution anatomical scaffold

We examined whether the organization of radial glia could provide additional criteria to delineate the pallial territories defined by genoarchitectonic markers. In other vertebrate models, it has been reported that certain boundaries, such as the pallial–subpallial border and several inter-rhombomeric limits, coincide with fasciculated bundles of radial glia that act as recognizable anatomical discontinuities (de Carlos et al. 1996; Neyt et al. 1997; Yoshida and Colman 2000; Trujillo et al. 2004; Delgado et al. 2005). These precedents justified exploring whether a comparable principle could be applied to the reptilian pallium.

In turtles, GFAP immunolabeling revealed regionally differentiated glial patterns, such as dense, rectilinear bundles in the dorsal and medial cortices; oblique, convergent fibers in the PT, defining a morphological domain consistent with its molecular identity; arcuate trajectories in the aLCx that allow discrimination of its dorsal and ventral subdivisions; and a compact radial bundle in the anterior DVR, compatible with its nuclear organization. These variations reflect a glial architecture that, to some extent, recapitulates the internal logic of each territory.

Nevertheless, its ability to delineate boundaries proved limited. In most domains, differences in fiber density, orientation, or trajectory did not generate sufficiently abrupt discontinuities to establish borders independently. Thus, in the reptilian pallium, radial glial cells provide a useful morphoarchitectonic framework for organizing and reinterpreting anatomy, but their resolving power is clearly insufficient without the support of broad panels of molecular markers and, when available, connectivity data, as has been demonstrated in other telencephalic systems (Garcia-Calero and Puelles 2020). Thus, while the expression of markers such as Ctip2, Satb1, and Satb2 allows several pallial territories to be delineated with relatively sharp borders, the partial overlap and changing combinations of these markers across regions suggest that pallial regionalization should not be interpreted as strictly discrete. Rather, it may involve both boundary-like transitions and graded molecular features, as proposed in recent comparative analyses (Moreau et al. 2021; Yamamoto et al. 2024).

### Progenitor diversity in sauropsids

Finally, mitotic labeling (PH3) at pre-laminar stages revealed that both turtles and geckos rely predominantly on apical radial glial cells (aRGCs) dividing at the ventricular surface, consistent with the ancestral neurogenic program described in non-mammalian vertebrates (De Juan and Borrell 2015; Montiel et al. 2016; Cárdenas et al. 2018). Although non-avian reptiles lack a well-developed subventricular zone (Charvet et al. 2009), turtles displayed sparse abventricular mitoses, indicating a minor population of BPs (Nomura et al. 2013a). These basal divisions were rare, showed no domain-specific differences, and persisted at low frequency even after lamination, suggesting a very limited amplifying potential insufficient to account for the variations in structures such as the PT. The absence of abventricular divisions in geckos aligns with interpretations proposing that lepidosaurs rely almost exclusively on apical progenitors (Nomura et al. 2013a; Cárdenas et al. 2018). Thus, the few BPs observed in turtles may represent the retention, or a modest elaboration, of an ancestral progenitor type rather than a basal progenitor expansion comparable to that seen in mammals (Reillo et al. 2011; Stahl et al. 2013; Coquand et al. 2024).

Because BP-like mitoses were observed only in turtles, we considered whether these cells could correspond to IPCs, defined by Eomes/Tbr2 expression and by amplifying divisions in the mammalian subventricular zone (Mihalas and Hevner 2017; Hevner 2019). However, the presence of IPCs in reptiles remains unresolved. Some studies have reported mitotic Tbr2^+^ cells in turtles (Clinton et al. 2014; Martínez-Cerdeño et al. 2016), whereas others, including the present study, have found no evidence of proliferative IPCs, although ouranalysis relies on a single M-phase marker (PH3) (Nomura et al. 2013a). If IPCs exist in chelonians, they are likely extremely rare or detectable only under specific experimental conditions. It is noteworthy that in birds the presence of BPs with high neurogenic potential has indeed been described; however, these progenitors are not homologous to mammalian IPCs, as they differ in their molecular profiles, proliferative dynamics, and their limited dependence on Eomes/Tbr2 (Nomura et al. 2016; Garcia-Calero and Puelles 2020). Consistent with this view, recent studies based on single-cell transcriptomics and lineage reconstruction in birds have failed to identify progenitor populations equivalent to proliferative mammalian-type IPCs (Rueda-Alaña et al. 2025; Zaremba et al. 2025). By contrast, several studies in anamniotes have identified IPC-like progenitor populations (Docampo-Seara et al. 2018; Deryckere et al. 2025; Quintana-Urzainqui et al. 2025), suggesting that this lineage may represent an ancestral vertebrate condition secondarily reduced or lost in sauropsids (García-Moreno and Molnár 2020). Under this scenario, mammals would have expanded and diversified this progenitor pool, whereas reptiles and birds retained a predominantly apical neurogenic program and, at least in turtles, only a sparse basal population not homologous to mammalian IPCs. Additionally, birds also expanded another BP population and in discrete pallial regions only, such as the embryonic DVR (Rueda-Alaña et al. 2025).

### Limitations and future perspectives

Although this study provides an unprecedented genoarchitectonic mapping of the reptilian pallium, several limitations should be acknowledged. First, the functional characterization of the identified domains remains largely indirect. While putative olfactory or thalamic roles are inferred from established connectivity patterns and comparative evidence, these functional assignments require direct validation through pathway-specific tracing, activity-dependent markers, or targeted neuronal activation and silencing approaches. In particular, a more precise inference of thalamo–pallial functional relationships will require systematic analyses of thalamic input specificity and their impact on local circuit activity across pallial subdivisions.

Second, a major unresolved challenge concerns the developmental linkage between embryonic germinal territories and their mature counterparts in the adult brain. Lineage-tracing studies are critically needed to directly connect defined progenitor domains with specific adult pallial regions and their corresponding neuronal populations, an approach that remains largely unexplored in reptiles. Without such developmental reconstructions, homology assessments based on adult molecular profiles alone remain necessarily incomplete.

Finally, the integration of lineage analyses with spatial transcriptomics, high-resolution connectivity mapping, and functional manipulations will be essential to bridge genoarchitectonic subdivisions with defined circuits and behavioral outputs. Such integrative strategies will enable comparative neuroanatomical frameworks to converge with functional and developmental neurobiology, ultimately advancing our understanding of the evolutionary origins and lineage-specific diversification of the vertebrate telencephalon.

## Supplementary Material

Supplementary_material_bhag131

## Data Availability

The data underlying this article are available in the article and in its online supplementary material.
