# Supplementary material for: Divergent structural trajectories for pallial regionalization within reptiles reveal conserved evolutionary blueprints
Source: Cereb Cortex. 2026 Sep 16;36(9):bhag131. doi: 10.1093/cercor/bhag131 (PMC13580228; doi:10.1093/cercor/bhag131)
Supplement: Supplementary file 1 [file supplementary_material_bhag131.zip › Supplementary Material.docx]

**Supplementary Materials**

**Divergent structural trajectories for pallial regionalization within reptiles reveal conserved evolutionary blueprints**

Sara Jiménez^1^, Ana Moreno-Cerdá^2^, Juan-Antonio Moreno-Bravo^2^, Nerea Moreno^3^ and Fernando García-Moreno^1,4,5*^.

^1^ Achucarro Basque Center for Neuroscience, Scientific Park of the University of the Basque Country (UPV/EHU), 48940, Leioa, Spain.

^2^ Instituto de Neurociencias, Consejo Superior de Investigaciones Científicas & Universidad Miguel Hernández, Sant Joan d’Alacant 03550, Spain.

^3^ Department of Cell Biology and Histology, Faculty of Biological Sciences, University Complutense, Madrid, Spain.

^4^ Department of Neuroscience, Faculty of Medicine and Odontology, UPV/EHU, Barrio Sarriena s/n, 48940 Leioa, Bizkaia, Spain.

^5^ IKERBASQUE Foundation, María Díaz de Haro 3, 6th Floor, 48013 Bilbao Spain.

* Corresponding authorship: [fernando.garcia-moreno@achucarro.org](mailto:fernando.garcia-moreno@achucarro.org)

**Supplementary Figure and Video captions**

**Figure S1. Comparative cytoarchitecture of the dorsal pallium in gecko and turtle.**
Nissl-stained coronal sections comparing the rostrocaudal organization of the dorsal pallium in Paroedura picta and Trachemys scripta elegans. **(A, C, E, G)** In gecko, the dorsal cortex displays a laminar organization, with identifiable L2 and L3 domains, but no clearly distinguishable nuclear territory comparable to the turtle pallial thickening. **(B, D, F, H)** In turtle, a prominent pallial thickening (PT) is evident at anterior and intermediate levels, adjacent to the dorsal cortex. At more caudal levels, the PT is no longer apparent and the posterior dorsal cortex (pDCx) occupies the corresponding dorsal pallial region. These observations support the interpretation that, in gecko, the region occupying a comparable topological position to the turtle PT is incorporated within the laminar organization of the dorsal cortex rather than forming a separate PT-like nuclear domain. This interpretation is based on the results observed in Figure 3. **Abbreviations:** aDCx, anterior dorsal cortex; DCx, dorsal cortex; L2, layer 2; L3, layer 3; pDCx, posterior dorsal cortex; PT, pallial thickening. **Scale bars:** 200 µm.

**Video S1. Three-dimensional reconstructions of pallial subdivisions in turtle.** Reconstructed volumes of the forebrain of Trachemys scripta elegans obtained by light sheet fluorescence microscopy. The expression patterns of Prox1, Satb1, Satb2 and Ctip2 were used to segment and delineate pallial regions. The movie shows sequential rostrocaudal reconstructions, illustrating the spatial topology and molecularly defined boundaries of each pallial subdivision. **Abbreviations:** aDVR, anterior part of the dorsal ventricular ridge; DCx, dorsal cortex; DMCx, dorsomedial cortex; LCx, lateral cortex; MCx, medial cortex; pDVR, posterior part of the dorsal ventricular ridge; PT, pallial thickening. **Scale bar:** 1000 µm.

**Video S2. Three-dimensional reconstructions of pallial subdivisions in gecko.** Reconstructed volumes of the forebrain of Paroedura picta obtained by light sheet fluorescence microscopy. The expression patterns of Prox1, Satb1, Satb2 and Ctip2 were used to segment and delineate pallial regions. The movie shows sequential rostrocaudal reconstructions, illustrating the spatial topology and molecularly defined boundaries of each pallial subdivision. **Abbreviations:** aDVR, anterior part of the dorsal ventricular ridge; DCx, dorsal cortex; DMCx, dorsomedial cortex; LCx, lateral cortex; MCx, medial cortex; pDVR, posterior part of the dorsal ventricular ridge. **Scale bar:** 500 µm.

**Video S3. Rostrocaudal orthoslice views of turtle pallial subdivisions obtained by 3D light-sheet imaging.** Expression of Prox1, Satb1, Satb2 and Ctip2 is shown from rostral to caudal regions of the forebrain of Trachemys scripta elegans, obtained by light sheet fluorescence microscopy. Pallial markers were used to segment and delineate the medial, dorsomedial, dorsal and lateral cortex and the dorsal ventricular ridge (DVR). The movie illustrates the topology and molecularly defined boundaries of each pallial subdivision. **Abbreviations:** aDVR, anterior part of the dorsal ventricular ridge; DCx, dorsal cortex; DMCx, dorsomedial cortex; LCx, lateral cortex; MCx, medial cortex; pDVR, posterior part of the dorsal ventricular ridge; PT, pallial thickening. **Scale bar:** 1000 µm.

**Video S4. Rostrocaudal orthoslice views of gecko pallial subdivisions obtained by 3D light-sheet imaging.** Expression of Prox1, Satb1, Satb2 and Ctip2 is shown from rostral to caudal regions of the forebrain of Paroedura picta, obtained by light sheet fluorescence microscopy. Pallial markers were used to segment and delineate the medial, dorsomedial, dorsal and lateral, and the dorsal ventricular ridge. The movie illustrates the topology and molecularly defined boundaries of each pallial subdivision. **Abbreviations:** aDVR, anterior part of the dorsal ventricular ridge; DCx, dorsal cortex; DMCx, dorsomedial cortex; LCx, lateral cortex; MCx, medial cortex; pDVR, posterior part of the dorsal ventricular ridge. **Scale bar:** 500 µm.
